# Association between dietary choline intake and odds of preeclampsia: a case–control study

**DOI:** 10.3389/fnut.2025.1703117

**Published:** 2025-11-04

**Authors:** Junhua Zhu, Yacong Bo, Ruixue Ma, Ziwei Jiang, Jiahan Wang, Zheng Yuan, Xianlan Zhao, Yuan Cao, Dandan Duan, Weifeng Dou, Yanhua Liu, Quanjun Lyu

**Affiliations:** ^1^Department of Nutrition, The First Affiliated Hospital of Zhengzhou University, Zhengzhou, China; ^2^Department of Nutrition and Food Hygiene, College of Public Health, Zhengzhou University, Zhengzhou, China; ^3^Department of Obstetrics, The First Affiliated Hospital of Zhengzhou University, Zhengzhou, China; ^4^The Third Affiliated Hospital of Zhengzhou University, Zhengzhou, China; ^5^Department of Public Health, Zhengzhou Shuqing Medical College, Zhengzhou, China

**Keywords:** dietary choline, choline subtypes, choline sources, preeclampsia, case–control study

## Abstract

**Background:**

Preeclampsia (PE) is a leading cause of maternal and perinatal morbidity and mortality. Choline, essential in one-carbon metabolism and vascular function, may influence placental health. We examined associations of total, subtype-, and source-specific dietary choline with PE odds in Chinese women.

**Methods:**

We conducted a 1:1 matched case–control study of 982 pregnant women (491 PE cases; 491 controls) in Zhengzhou, China. Dietary intake over the preceding three months was assessed using a validated semi-quantitative food-frequency questionnaire. Conditional logistic regression calculated odds ratios (ORs) and 95% confidence intervals (CIs) for total choline, lipid- vs. water-soluble forms, and animal- vs. plant-derived sources, adjusting for covariates. Restricted cubic splines explored possible non-linear dose–response associations.

**Results:**

Among 982 participants (491 PE cases; 491 controls), mean total choline intake was 335.8 mg/day, with eggs contributing 42.5%. In multivariable-adjusted models, compared with the lowest quartile, those in the highest quartile of total choline intake had 58% lower odds of PE (OR = 0.42; 95% CI, 0.26–0.68), with similar associations for lipid- (0.33; 0.22–0.48) and water-soluble forms (0.37; 0.25–0.54). Both animal- (0.43; 0.30–0.63) and plant-derived choline (0.31; 0.21–0.46) were protective, while their intake ratio was not. Each additional 25 g/day of egg (~half an egg) was linked to an 11% lower PE odds.

**Conclusion:**

Higher habitual dietary choline intakes from animal and plant sources were independently associated with significantly lower odds of PE, suggesting that adequate, source-diverse choline intake in early pregnancy may offer a practical dietary strategy for PE prevention.

## 1 Introduction

Preeclampsia (PE) remains a major cause of maternal and perinatal morbidity and mortality worldwide, affecting approximately 2–8% of pregnancies and contributing to around 46,000 maternal deaths annually (1–3). Despite notable advances in obstetric care, its incidence is increasing in many regions, including China—largely driven by rising maternal age and a growing burden of cardiometabolic risk factors (4, 5). Beyond its acute complications during pregnancy, PE also confers substantial long-term risks for cardiovascular and metabolic disorders in both mothers and their offspring (2, 6–9), underscoring the urgent need to identify modifiable risk factors.

Emerging evidence implicates abnormal placentation, endothelial dysfunction, and heightened oxidative stress as key mechanisms in PE pathogenesis (10, 11). Among potentially modifiable factors, nutritional exposures have received substantial attention (12). Choline—a conditionally essential nutrient central to one-carbon metabolism, methylation reactions, and membrane phospholipids—has received comparatively less focus despite mounting data implicating roles in placental function and vascular health (13, 14). In many high-income populations, usual maternal choline intake often falls short of pregnancy-appropriate recommendations (15, 16). Although inadequate intake has been linked primarily to fetal/neurodevelopmental concerns (16, 17), evidence connecting low maternal choline intake or status to maternal clinical outcomes—particularly PE—remains limited.

Experimental and epidemiological studies indicate that inadequate maternal choline intake may impair fetal neurodevelopment, increase the risk of neural tube defects, and adversely affect pregnancy outcomes (16, 18). Yet, the relationship between dietary choline intake and PE risk remains poorly understood (13), hindered by methodological limitations in the existing literature. Large-scale prospective studies are lacking, and most prior work has relied on cross-sectional or retrospective designs, limiting causal inference (19). Moreover, little attention has been paid to the heterogeneity of choline subtypes or their distinct dietary sources, and few studies have considered the influence of population-specific dietary patterns (13). Notably, recent Mendelian randomization analyses implicate circulating choline metabolites in PE development (20), but direct nutritional epidemiologic evidence is needed to clarify these associations and inform clinical practice.

To address these gaps, we conducted a matched case–control study in China to examine the associations between total dietary choline intake, specific chemical subtypes, and major dietary sources with the odds of PE. To our knowledge, this is the first comprehensive investigation of dietary choline and PE odds in an Asian population, offering novel insights with potential implications for targeted nutritional strategies and policy recommendations.

## 2 Methods

### 2.1 Study design and participants

We conducted a 1:1 matched case–control study nested within a maternal nutrition surveillance program at the First Affiliated Hospital of Zhengzhou University, China (March 2016–June 2019), designed to investigate the association between dietary choline intake and the odds of preeclampsia (PE).

PE diagnosis followed the 2015 Chinese guidelines (21), requiring new-onset hypertension (≥140/90 mmHg) after 20 weeks’ gestation accompanied by either proteinuria (≥0.3 g/24 h) or evidence of organ dysfunction. Eligible participants were pregnant women aged 18–45 years with singleton pregnancies and no history of gestational hypertension. Each case was individually matched to a control based on maternal age (±3 years), gestational age (±1 week), and gestational diabetes mellitus (GDM) status. Exclusion criteria included chronic hypertension, diabetes, renal or psychiatric disease, and implausible energy intake (<500 or >5,000 kcal/day). Of the 1,218 women screened, 491 matched pairs (*n* = 982) were included in the final analysis (Supplementary Figure 1). Power calculations indicated that the sample size provided 80% power (*α* = 0.05) to detect an odds ratio of 0.50 for high versus low choline intake (22). The study was approved by the Ethics Committee of the First Affiliated Hospital of Zhengzhou University (No. Scientific research-2016-LW-34), and all participants provided written informed consent.

### 2.2 Assessment of dietary choline

Dietary intake was assessed during face-to-face interviews using a validated 79-item semi-quantitative food-frequency questionnaire (FFQ) capturing usual intake over the three months preceding delivery. The FFQ’s accuracy for estimating energy and nutrient intakes has been previously confirmed (23, 24). For each food item, intake frequency (never, monthly, weekly, daily) and portion size were recorded, aided by a standardized color photo booklet to improve portion-size estimation.

Energy (kcal/day) and most nutrient intakes were calculated using the Chinese Food Composition Tables (25). As choline and betaine values were not available in this database, these nutrient contents were obtained from the USDA FoodData Central (U.S. Department of Agriculture, Agricultural Research Service) (26). Total dietary choline was calculated as the sum of phosphatidylcholine (PtdCho), sphingomyelin (SM), free choline, glycerophosphocholine (GPCho), and phosphocholine (PCho). Choline was further classified into lipid-soluble forms (PtdCho, SM) and water-soluble forms (free choline, GPCho, PCho), and into animal- and plant-derived sources based on food origin. Daily intakes of choline subtypes and betaine (mg/day) were estimated by multiplying the consumption of each food by its nutrient content per 100 g and summing across all foods.

To account for total energy intake, we additionally derived energy-adjusted choline (and subtypes) using the residual method (27); unadjusted values were used for descriptive analyses, and both unadjusted and energy-adjusted values were included in sensitivity analyses. Supplement use (e.g., folic acid, multivitamins) was recorded and included as covariates in multivariable analyses.

### 2.3 Assessment of preeclampsia

PE status was ascertained from medical records and confirmed by senior obstetricians according to the 2015 Chinese guidelines for hypertensive disorders of pregnancy. Diagnosis required new-onset hypertension—systolic blood pressure ≥140 mmHg or diastolic ≥90 mmHg—after 20 weeks’ gestation, plus either: (1) proteinuria (≥0.3 g/24 h, protein-to-creatinine ratio ≥0.3, or dipstick ≥ “+”), or (2) in the absence of proteinuria, evidence of maternal organ/system dysfunction (hepatic, renal, cardiovascular, respiratory, hematologic, neurologic, or placental–fetal involvement).

Blood pressure was measured twice, five minutes apart, with a calibrated automated sphygmomanometer; the mean value was used for classification. Urinary protein was measured using 24-h collections when feasible; otherwise, spot protein-to-creatinine ratio or dipstick tests were applied per guideline recommendations. All PE cases met these criteria, whereas controls remained normotensive with no evidence of organ dysfunction throughout pregnancy.

### 2.4 Assessment of covariates

Covariates were selected based on established links with dietary patterns and hypertensive disorders in pregnancy. The interviewer-administered structured questionnaire was used only for non-dietary covariates. Sociodemographic variables included maternal age (years), monthly household income (≤2,000; 2,001–4,000; 4,001–6,000; >6,000 yuan), educational attainment (middle school or below, high school or equivalent, college or above), and employment status (employed/unemployed). Lifestyle factors included smoking and alcohol use (ever/never), physical activity (MET-hours/day, continuous), and self-reported sleep quality (poor/moderate/good). Reproductive history variables included parity (0, 1, ≥2), menstrual regularity (yes/no), and gestational age (weeks, continuous). Pre-pregnancy BMI (kg/m^2^, continuous) and supplement use (folic acid, multivitamins) were self-reported at baseline. Dietary energy intake (kcal/day, continuous) was calculated from the FFQ, and the dietary assessment period was categorized by season (spring, summer, autumn, winter). Clinical covariates included GDM status (yes/no) and family history of hypertension (yes/no). Psychological distress was assessed with standardized Zung Self-Rating Anxiety Scale (28) (SAS) and Self-Rating Depression Scale (29) (SDS) scores.

### 2.5 Statistical analysis

Continuous variables are presented as means (standard deviations [SD]) and categorical variables as counts (percentages). Differences between PE cases and controls were assessed using Student’s t test or Chi-square test, as appropriate. For variables with <25% missing data, multiple imputation (five imputations) was performed, and pooled estimates were calculated using Rubin’s rules (30).

The primary analyses examined associations between total choline, individual choline compounds (free choline, PCho, PtdCho, GPCho, SM), and betaine intake and the odds of PE using conditional logistic regression for matched pairs. Additional analyses evaluated choline subcategories (lipid-soluble, water-soluble, lipid-to-water choline intake ratio), source-specific choline (animal-derived, plant-derived, animal-to-plant intake ratio), and daily egg intake—the primary dietary source of choline in this case–control study. For each exposure, intakes were categorized into quartiles according to the control group distribution (lowest quartile as reference). Trends across quartiles were tested by modeling the median intake of each quartile as a continuous variable. Associations per 1-SD increment in intake were also estimated. Odds ratios (ORs) and 95% confidence intervals (CIs) were derived from three models: Model 1 adjusted for maternal age, gestational age, and pre-pregnancy BMI; Model 2 additionally adjusted for household income, educational attainment, physical activity, employment status, smoking, alcohol use, and sleep quality; and Model 3 further adjusted for energy intake, assessment season, parity, GDM, menstrual regularity, family history of hypertension, and supplement use. Restricted cubic spline models were used to explore potential non-linear associations between choline-related exposures and the odds of PE.

Stratified and interaction analyses assessed effect modification within prespecified subgroups, with interaction *p* values derived from likelihood ratio tests (unadjusted for multiple comparisons). A series of sensitivity analyses were performed to test the robustness of findings: (1) analyses restricted to complete cases; (2) further adjustment for psychological distress (anxiety and depression scores); (3) exclusion of participants diagnosed with GDM; (4) analyses without adjustment for daily energy intake to examine associations irrespective of total energy; and (5) analyses using residual energy-adjusted choline intake.

## 3 Results

### 3.1 Baseline characteristics

**Figure 1:**
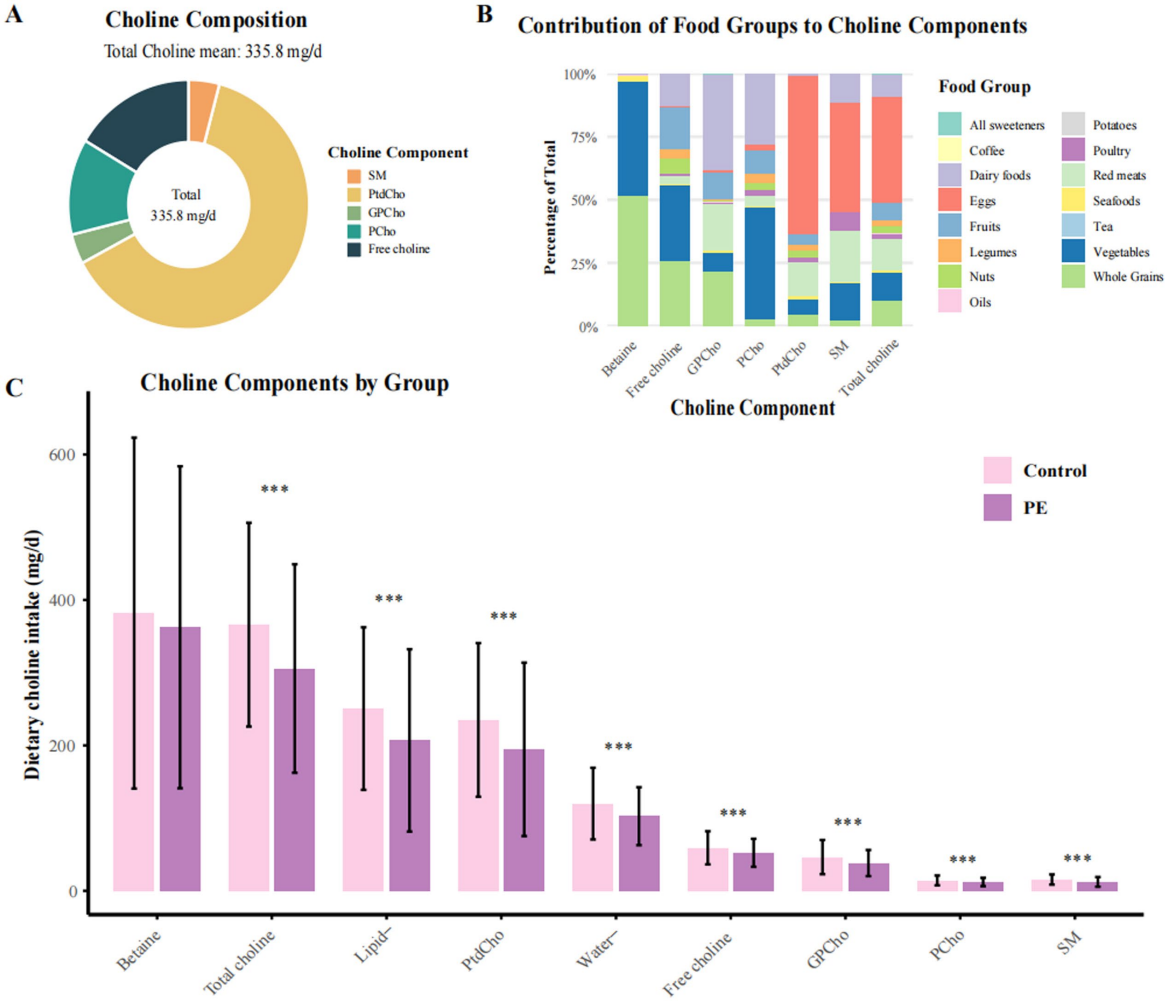
Distribution and food source composition of dietary choline and betaine intake among preeclampsia cases and controls. PE, preeclampsia; PtdCho, phosphatidylcholine; SM, sphingomyelin; GPCho, glycerophosphocholine; PCho, phosphocholine. **(A)** Composition of total dietary choline intake among all participants. In this study population, the average total choline intake was 335.1 mg/day. **(B)** Relative contributions of major food groups to total dietary choline and betaine intake. Eggs constituted the predominant dietary source of choline, accounting for 42.48% of total intake. **(C)** Comparative distribution of lipid-soluble and water-soluble choline, as well as betaine intake, between women with preeclampsia and controls. Lipid-soluble choline includes PtdCho and SM; water-soluble choline includes free choline, GPCho, and PCho. Values are presented as means, with error bars denoting standard deviations (± SD). *p < 0.05, **p < 0.01, ***p < 0.001 for group comparisons.

A total of 982 pregnant women (mean age: 31.1 ± 5.0 years) were included, comprising 491 preeclampsia (PE) cases and 491 matched controls. The mean (SD) total dietary choline intake was 335.8 (144.8) mg/day (range: 55.8–954.6 mg/day), with higher choline intakes generally observed among women with greater total energy and nutrient consumption (Table 1; Figure 1A; Supplementary Table 1). In terms of choline composition, lipid-soluble forms—primarily phosphatidylcholine (PtdCho) and sphingomyelin (SM)—accounted for 67.3% of total choline intake, while water-soluble forms (free choline, glycerophosphocholine [GPCho], and phosphocholine [PCho]) contributed the remaining 32.7% (Figure 1B; Supplementary Table 2). When examining dietary sources, eggs were the predominant dietary source, providing 42.5% of total choline, followed by red meat (12.9%), vegetables (11.1%), whole grains (10.0%), and dairy products (8.9%). The proportional contributions of these sources were similar between PE cases and controls.

**Table 1:** Baseline characteristics of participants across quartiles of total dietary choline intake.

Characteristics	Total (*n* = 982)	Q1 (*n* = 329)	Q2 (*n* = 256)	Q3 (*n* = 204)	Q4 (*n* = 193)	*P* value
Maternal age, years	31.05 ± 5.02	31.05 ± 5.05	31.29 ± 5.03	30.52 ± 4.71	31.30 ± 5.29	0.35
Gestational age, weeks	34.19 ± 2.84	33.80 ± 2.83	34.38 ± 2.88	34.35 ± 2.72	34.44 ± 2.90	**0.02**
Pre-pregnancy BMI, kg/m^2^	23.11 ± 3.63	23.59 ± 4.15	22.95 ± 3.24	22.67 ± 3.14	22.95 ± 3.56	**0.02**
Physical activity (MET- h/d)	26.87 ± 4.31	26.97 ± 4.29	26.74 ± 4.08	26.67 ± 4.09	27.07 ± 4.86	0.74
Daily energy intake (kcal/day)	1920.00 ± 547.69	1602.16 ± 376.90	1834.42 ± 386.05	2034.25 ± 485.04	2454.58 ± 599.38	**<0.001**
Anxiety score	38.25 ± 6.97	37.71 ± 6.42	37.74 ± 7.51	38.40 ± 6.74	39.69 ± 7.20	**<0.01**
Depression score	39.62 ± 8.95	39.98 ± 8.51	38.61 ± 9.54	39.06 ± 8.57	40.96 ± 9.14	**0.03**
Income (Yuan/month)						0.08
≤2,000	128 (13.03)	59 (17.93)	28 (10.94)	16 (7.84)	25 (12.95)	
2,001–4,000	523 (53.26)	171 (51.98)	137 (53.52)	114 (55.88)	101 (52.33)	
4,001–6,000	180 (18.33)	60 (18.24)	47 (18.36)	38 (18.63)	35 (18.13)	
>6,000	151 (15.38)	39 (11.85)	44 (17.19)	36 (17.65)	32 (16.58)	
Educational attainment, %						**<0.001**
Middle school or below	405 (41.24)	167 (50.76)	99 (38.67)	65 (31.86)	74 (38.34)	
High school or equivalent	184 (18.74)	65 (19.76)	48 (18.75)	32 (15.69)	39 (20.21)	
College or above	393 (40.02)	97 (29.48)	109 (42.58)	107 (52.45)	80 (41.45)	
Employment status, %						0.31
Employed	332 (33.81)	100 (30.40)	94 (36.72)	75 (36.76)	63 (32.64)	
Unemployed	650 (66.19)	229 (69.60)	162 (63.28)	129 (63.24)	130 (67.36)	
Smoking status, %						0.70
Ever	149 (15.17)	49 (14.89)	35 (13.67)	36 (17.65)	29 (15.03)	
Never	833 (84.83)	280 (85.11)	221 (86.33)	168 (82.35)	164 (84.97)	
Drinking status, %						0.55
Ever	20 (2.04)	7 (2.13)	3 (1.17)	4 (1.96)	6 (3.11)	
Never	962 (97.96)	322 (97.87)	253 (98.83)	200 (98.04)	187 (96.89)	
Sleep quality, %						0.99
Poor	264 (26.88)	87 (26.44)	70 (27.34)	57 (27.94)	50 (25.91)	
Moderate	396 (40.33)	133 (40.43)	107 (41.80)	80 (39.22)	76 (39.38)	
Good	322 (32.79)	109 (33.13)	79 (30.86)	67 (32.84)	67 (34.72)	
Survey season, %						0.20
Spring	267 (27.19)	90 (27.36)	63 (24.61)	71 (34.80)	43 (22.28)	
Summer	211 (21.49)	68 (20.67)	56 (21.88)	37 (18.14)	50 (25.91)	
Autumn	275 (28.00)	92 (27.96)	73 (28.52)	58 (28.43)	52 (26.94)	
Winter	229 (23.32)	79 (24.01)	64 (25.00)	38 (18.63)	48 (24.87)	
Parity, %						0.22
0	365 (37.17)	128 (38.91)	89 (34.77)	78 (38.24)	70 (36.27)	
1	435 (44.30)	128 (38.91)	121 (47.27)	96 (47.06)	90 (46.63)	
≥2	182 (18.53)	73 (22.19)	46 (17.97)	30 (14.71)	33 (17.10)	
Preeclampsia, %						**<0.001**
No	491 (50.00)	123 (37.39)	123 (48.05)	122 (59.80)	123 (63.73)	
Yes	491 (50.00)	206 (62.61)	133 (51.95)	82 (40.20)	70 (36.27)	
GDM, %	136 (13.85)	33 (10.03)	35 (13.67)	34 (16.67)	34 (17.62)	0.05
Menstrual regularity, %	909 (92.57)	300 (91.19)	234 (91.41)	194 (95.10)	181 (93.78)	0.29
Family history of hypertension, %	285 (29.02)	101 (30.70)	80 (31.25)	55 (26.96)	49 (25.39)	0.44
Dietary intake						
Egg (g/day)	54.65 ± 34.62	24.60 ± 17.98	50.96 ± 18.53	69.75 ± 26.73	94.81 ± 30.48	**<0.001**
Vegetable (g/day)	355.83 ± 172.79	286.84 ± 122.68	353.14 ± 155.33	371.51 ± 160.08	460.41 ± 219.74	**<0.001**
Fruit (g/day)	363.84 ± 275.23	291.00 ± 221.67	313.90 ± 170.23	425.19 ± 309.37	489.39 ± 362.15	**<0.001**
Protein (g/day)	66.32 ± 23.00	47.86 ± 12.28	62.99 ± 13.65	73.87 ± 14.21	94.21 ± 23.29	**<0.001**
Fat (g/day)	74.77 ± 25.72	61.80 ± 19.12	70.89 ± 20.50	78.22 ± 21.57	98.38 ± 28.72	**<0.001**
Carbohydrate (g/day)	252.40 ± 82.35	219.20 ± 63.32	242.57 ± 62.49	266.40 ± 85.43	307.22 ± 98.25	**<0.001**
Total choline (mg/day)	335.84 ± 144.82	195.95 ± 50.71	307.64 ± 23.67	393.32 ± 27.04	550.96 ± 128.92	**<0.001**
Betaine (mg/day)	372.16 ± 231.62	324.02 ± 173.29	359.90 ± 231.06	403.97 ± 239.65	436.84 ± 285.87	**<0.001**
Lipid-soluble choline (mg/day)	226.02 ± 120.02	116.27 ± 45.51	204.63 ± 29.46	271.36 ± 43.83	393.57 ± 123.37	**<0.001**
Water-soluble choline (mg/day)	109.82 ± 45.18	79.68 ± 24.05	103.01 ± 24.72	121.97 ± 33.76	157.39 ± 58.11	**<0.001**
Animal−derived choline (mg/day)	180.82 ± 115.17	84.27 ± 46.37	164.14 ± 43.09	219.04 ± 60.08	327.12 ± 135.66	**<0.001**
Plant−derived choline (mg/day)	155.02 ± 67.52	111.68 ± 31.64	143.50 ± 41.69	174.28 ± 54.94	223.84 ± 86.79	**<0.001**
Free choline (mg/day)	54.96 ± 21.07	42.16 ± 12.39	51.79 ± 13.39	59.98 ± 16.98	75.69 ± 26.67	**<0.001**
Phosphocholine (mg/day)	13.08 ± 6.28	9.07 ± 3.28	12.35 ± 3.68	14.50 ± 4.69	19.37 ± 8.48	**<0.001**
Glycerophosphocholine (mg/day)	41.78 ± 21.15	28.45 ± 11.30	38.87 ± 11.66	47.49 ± 17.05	62.34 ± 28.35	**<0.001**
Phosphatidylcholine (mg/day)	212.24 ± 113.75	108.85 ± 43.24	192.02 ± 29.05	254.83 ± 42.94	370.30 ± 118.11	**<0.001**
Sphingomyelin (mg/day)	13.78 ± 6.95	7.42 ± 2.98	12.61 ± 2.20	16.52 ± 3.14	23.27 ± 6.71	**<0.001**
Folic acid supplement, %	792 (80.65)	254 (77.20)	207 (80.86)	172 (84.31)	159 (82.38)	0.20
Multivitamin supplement, %	128 (13.03)	44 (13.37)	38 (14.84)	23 (11.27)	23 (11.92)	0.67

Q, quartile; BMI, body mass index; GDM, gestational diabetes mellitus.

Quartile cut-offs for total dietary total choline intake were defined based on the sample distribution as follows: Q1: 55.8–210.3 mg/day; Q2: 210.3–288.3 mg/day; Q3: 288.3–375.0 mg/day; Q4: 375.0–954.6 mg/day. The corresponding median intake for each quartile was 161.6 mg/day (Q1), 254.8 mg/day (Q2), 326.5 mg/day (Q3), and 454.6 mg/day (Q4). Each quartile comprised approximately 25% of the study population. Continuous variables are presented as mea*n* ± standard deviation (SD), and categorical variables are expressed as number [percentage (%)]. The proportion of missing data was as follows: monthly household income (5.6%), educational attainment (0.1%), smoking status (0.1%), alcohol consumption (0.1%), menstrual regularity (0.41%), season of survey (1.12%), and family history of hypertension (0.81%). All other variables, including maternal age, gestational age, pre-pregnancy BMI, physical activity, daily energy intake, anxiety and depression scores, employment status, sleep quality, parity, GDM, and use of folic acid or multivitamin supplements, were complete with no missing values.

Two-sided *p* values are presented without adjustment for multiple comparisons, with *p* values below 0.001 reported as <0.001. Bold values indicating statistical significance, *p* < 0.05).

Across intake quartiles, dietary and sociodemographic gradients were evident. Participants in the highest quartile of total choline intake (Q4, ≥375.0 mg/day) consumed substantially more daily energy (mean 2,454.6 kcal), egg consumption (94.8 g/day), and animal-derived choline (327.1 mg/day) than those in the lowest quartile (Q1, ≤210.3 mg/day; 1,602.2 kcal, 24.6 g/day, and 84.3 mg/day, respectively). Moreover, higher choline intake was also associated with higher educational attainment, greater household income, and more diverse dietary patterns. Compared with controls, participants with PE had significantly lower total choline intake (305.7 vs. 366.0 mg/day), including both lipid- and water-soluble forms, and consumed fewer eggs and other animal-derived foods (Figure 1C). Additionally, PE cases also exhibited higher pre-pregnancy BMI, a greater prevalence of family history of hypertension, and poorer sleep quality. Correlations were strongest between PtdCho and SM (*r* = 0.89), with moderate correlations among water-soluble subtypes (Supplementary Figure 2).

### 3.2 Dietary choline, betaine intake, and odds of preeclampsia

**Figure 2:**
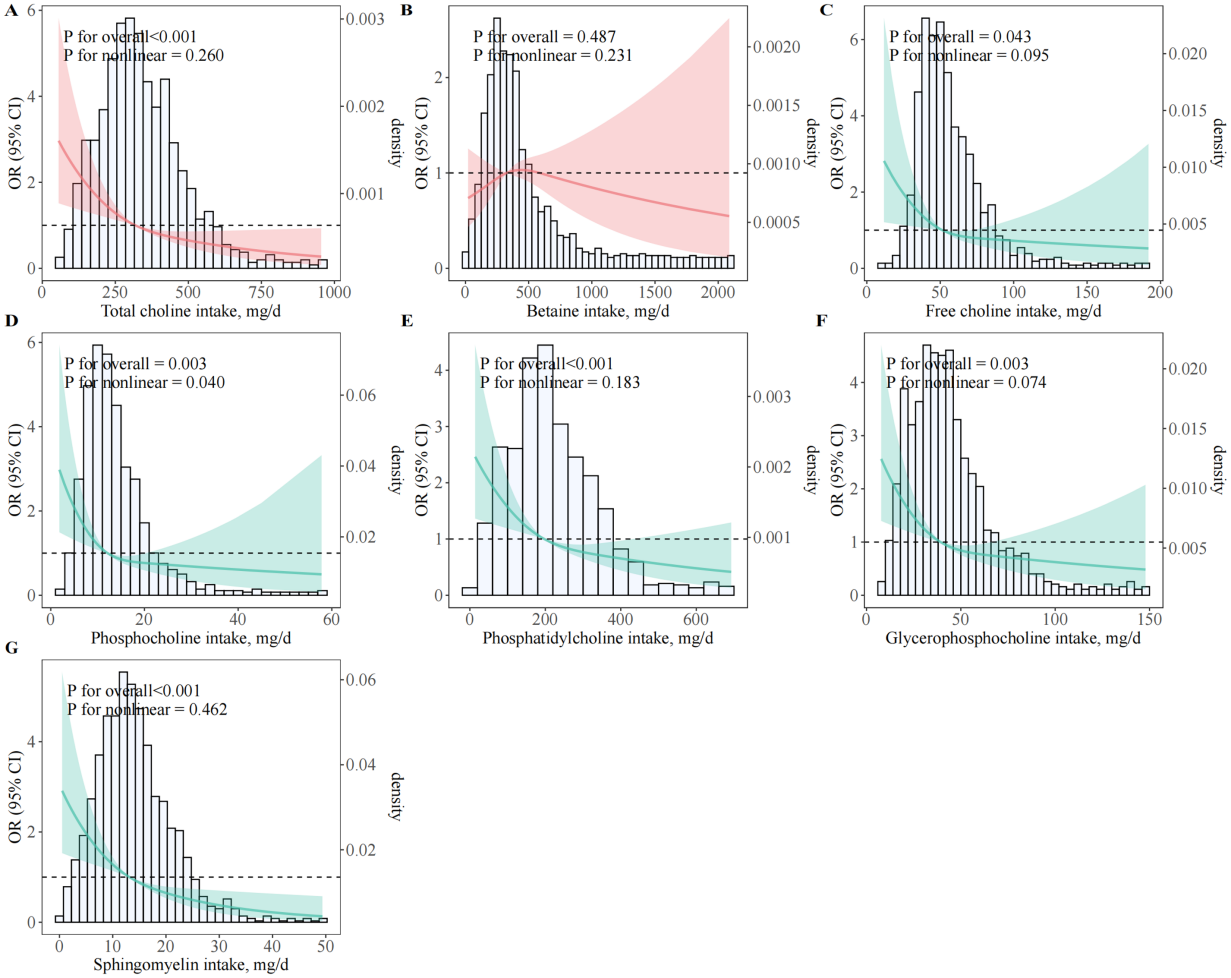
Dose–response associations between choline and betaine intake and odds of preeclampsia. **(A)**Total choline; **(B)** Betaine; **(C)** Free choline; **(D)** PCho; **(E)** PtdCho; **(F)** GPCho; **(G)** SM; OR, odds ratio; CI, confidence interval; BMI, body mass index; GDM, gestational diabetes mellitus; PCho, phosphocholine; GPCho, glycerophosphocholine; PtdCho, phosphatidylcholine; SM, sphingomyelin. Restricted cubic spline regression models illustrating multivariable-adjusted dose–response relationships between various forms of dietary choline and betaine intake and the odds of PE among pregnant women. All models were adjusted for maternal age (years), gestational age at survey (weeks), pre-pregnancy BMI (kg/m^2^), monthly household income, educational attainment, physical activity (MET-hours/day), employment status, smoking status, alcohol consumption, sleep quality, daily energy intake (kcal/day), season of dietary assessment, parity, GDM, menstrual regularity, family history of hypertension, and supplement use (folic acid and multivitamins).

Higher total dietary choline intake was strongly associated with lower odds of PE (Table 2; Figure 2A). In fully adjusted models, women in Q4 had 58% lower odds of PE compared with those in Q1 (95% CI, 0.26–0.68; *P*_trend_ < 0.001). Each 1-SD increment in total choline intake corresponded to a 31% lower odds of PE (95% CI, 0.57–0.84). These inverse associations were consistent across sequential models adjusting for sociodemographic, lifestyle, dietary, and reproductive factors (Model 1–Model 3). Restricted cubic spline analyses confirmed a significant linear inverse association (*P*_overall_ < 0.001; *P*_nonlinear_ = 0.260), with no evidence of a threshold effect (Figure 2A).

**Table 2:** Associations of total dietary choline and betaine intake with odds of preeclampsia.

Dietary choline	Cases/controls	Intake (mg/day)	Model 1		Model 2		Model 3	
			OR (95% CI)	*P* value	OR (95% CI)	*P* value	OR (95% CI)	*P* value
Total choline intake								
Q1	206/123	161.55 (55.82–210.32)	1 (reference)		1 (reference)		1 (reference)	
Q2	133/123	254.81 (210.33–288.28)	0.7 (0.50, 0.98)	0.04	0.74 (0.52, 1.04)	0.08	0.79 (0.54, 1.14)	0.21
Q3	82/122	326.5 (288.29–374.99)	0.43 (0.30, 0.62)	<0.001	0.46 (0.32, 0.67)	<0.001	0.54 (0.36, 0.82)	0.004
Q4	70/123	454.62 (>375.00)	0.36 (0.25, 0.53)	<0.001	0.37 (0.25, 0.54)	<0.001	0.42 (0.26, 0.68)	<0.001
*P* for trend				<0.001		<0.001		<0.001
Per SD increment			0.65 (0.56, 0.75)	<0.001	0.66 (0.57, 0.76)	<0.001	0.69 (0.57, 0.84)	<0.001
Free choline								
Q1	192/123	34.27 (11.68–38.98)	1 (reference)		1 (reference)		1 (reference)	
Q2	110/123	43.13 (38.99–48.26)	0.57 (0.41, 0.81)	0.001	0.59 (0.42, 0.84)	0.003	0.62 (0.43, 0.88)	0.01
Q3	116/122	54.52 (48.27–61.36)	0.61 (0.43, 0.86)	0.004	0.63 (0.45, 0.89)	0.01	0.65 (0.46, 0.93)	0.02
Q4	73/123	74.29 (>61.36)	0.38 (0.26, 0.55)	<0.001	0.39 (0.27, 0.57)	<0.001	0.39 (0.27, 0.57)	<0.001
*P* for trend				<0.001		<0.001		<0.001
Per SD increment			0.71 (0.62, 0.81)	<0.001	0.72 (0.62, 0.83)	<0.001	0.72 (0.62, 0.83)	<0.001
Phosphocholine								
Q1	204/123	6.73 (1.84–8.43)	1 (reference)		1 (reference)		1 (reference)	
Q2	115/123	9.5 (8.44–11.14)	0.59 (0.42, 0.83)	0.003	0.64 (0.45, 0.91)	0.01	0.69 (0.48, 1.01)	0.06
Q3	103/122	12.71 (11.15–14.75)	0.55 (0.39, 0.78)	<0.001	0.57 (0.40, 0.82)	0.002	0.56 (0.38, 0.85)	0.01
Q4	69/123	17.95 (>14.75)	0.36 (0.25, 0.52)	<0.001	0.39 (0.26, 0.57)	<0.001	0.45 (0.27, 0.73)	0.001
*P* for trend				<0.001		<0.001		<0.001
Per SD increment			0.69 (0.60, 0.80)	<0.001	0.71 (0.61, 0.83)	<0.001	0.75 (0.61, 0.92)	0.01
Glycerophosphocholine								
Q1	200/123	19.72 (7.78–25.36)	1 (reference)		1 (reference)		1 (reference)	
Q2	122/123	30.58 (25.37–35.95)	0.64 (0.46, 0.90)	0.01	0.69 (0.49, 0.98)	0.04	0.71 (0.49, 1.04)	0.08
Q3	100/122	41.23 (35.96–47.40)	0.52 (0.36, 0.74)	<0.001	0.55 (0.38, 0.79)	0.001	0.59 (0.39, 0.89)	0.01
Q4	69/123	58.52 (>47.40)	0.38 (0.26, 0.56)	<0.001	0.4 (0.27, 0.59)	<0.001	0.5 (0.30, 0.83)	0.01
*P* for trend				<0.001		<0.001		0.004
Per SD increment			0.66 (0.57, 0.77)	<0.001	0.68 (0.58, 0.79)	<0.001	0.72 (0.58, 0.90)	0.003
Phosphatidylcholine								
Q1	229/123	80.32 (13.96–117.80)	1 (reference)		1 (reference)		1 (reference)	
Q2	90/123	157.13 (117.81–178.52)	0.42 (0.30, 0.60)	<0.001	0.45 (0.31, 0.64)	<0.001	0.5 (0.34, 0.73)	<0.001
Q3	101/122	209.11 (178.53–246.02)	0.47 (0.33, 0.66)	<0.001	0.5 (0.35, 0.71)	<0.001	0.55 (0.37, 0.80)	0.002
Q4	71/123	312.81 (>246.02)	0.33 (0.23, 0.48)	<0.001	0.33 (0.23, 0.49)	<0.001	0.4 (0.26, 0.61)	<0.001
*P* for trend				<0.001		<0.001		<0.001
Per SD increment			0.69 (0.59, 0.79)	<0.001	0.7 (0.60, 0.80)	<0.001	0.76 (0.64, 0.89)	<0.001
Sphingomyelin								
Q1	220/123	5.62 (0.51–7.90)	1 (reference)		1 (reference)		1 (reference)	
Q2	123/123	9.7 (7.91–11.65)	0.6 (0.43, 0.84)	0.003	0.63 (0.45, 0.89)	0.01	0.63 (0.43, 0.91)	0.01
Q3	92/122	13.5 (11.66–15.60)	0.45 (0.32, 0.64)	<0.001	0.48 (0.33, 0.69)	<0.001	0.55 (0.37, 0.81)	0.003
Q4	56/123	19.34 (>15.60)	0.27 (0.18, 0.40)	<0.001	0.28 (0.19, 0.41)	<0.001	0.28 (0.17, 0.44)	<0.001
*P* for trend				<0.001		<0.001		<0.001
Per SD increment			0.61 (0.53, 0.70)	<0.001	0.62 (0.53, 0.72)	<0.001	0.64 (0.54, 0.77)	<0.001
Betaine								
Q1	121/123	165.67 (20.69–226.46)	1 (reference)		1 (reference)		1 (reference)	
Q2	157/124	270.82 (226.47–316.22)	1.22 (0.86, 1.73)	0.27	1.19 (0.83, 1.70)	0.35	1.38 (0.94, 2.03)	0.10
Q3	108/121	374.71 (316.23–432.21)	0.89 (0.62, 1.29)	0.55	0.86 (0.59, 1.25)	0.43	1.12 (0.74, 1.68)	0.60
Q4	105/123	587.12 (>432.21)	0.79 (0.54, 1.14)	0.21	0.75 (0.51, 1.10)	0.14	1.18 (0.76, 1.83)	0.47
*P* for trend				0.08		0.05		0.71
Per SD increment			0.89 (0.78, 1.02)	0.09	0.88 (0.77, 1.00)	0.05	1 (0.86, 1.17)	0.96

Q, quartile; OR, odds ratio; CI, confidence interval; BMI, body mass index; GDM, gestational diabetes mellitus.

Multivariable logistic regression models were used to estimate ORs and 95% CIs for the association between quartiles of individual choline subtypes and betaine intake and the odds of preeclampsia. The lowest quartile (Q1) was used as the reference group. P for trend was derived by modeling the median value of each quartile as a continuous variable. Per standard deviation (SD) increment estimates represent the change in odds of preeclampsia per one SD increase in the intake values.

Model 1 was adjusted for maternal age (years), gestational age at survey (weeks), and pre-pregnancy BMI (kg/m^2^).

Model 2 was further adjusted for socioeconomic and lifestyle factors, including monthly household income, educational attainment, physical activity (MET-hours/day), employment status, smoking status, alcohol consumption, and sleep quality.

Model 3 was additionally adjusted for dietary and reproductive variables, including daily energy intake (kcal/day), season of dietary assessment, parity, GDM, menstrual regularity, family history of hypertension, and supplement use (folic acid and multivitamins).

Two-sided *P* values are presented without adjustment for multiple comparisons, with *P* values below 0.001 reported as <0.001.

Similar inverse associations were observed for all individual choline subtypes (free choline, PCho, PtdCho, GPCho, and SM), with women in the highest quartile of each subtype consistently exhibiting markedly lower odds of PE compared to those in the lowest quartile. Fully adjusted ORs (95% CI) for the highest versus lowest quartile were: free choline (0.39; 95% CI, 0.27–0.57), PCho (0.45; 95% CI, 0.27–0.73), GPCho (0.50; 95% CI, 0.30–0.83), PtdCho (0.40; 95% CI, 0.26–0.61), and SM (0.28; 95% CI, 0.17–0.44); all *P*_trend_ ≤ 0.004 (Table 2; Figures 2C–G). By contrast, no significant association was observed for dietary betaine in any model (OR_Q4 versus Q1_ = 1.18; 95% CI, 0.76–1.83; *P*_trend_ = 0.71) (Table 2; Figure 2B).

**Figure 3:**
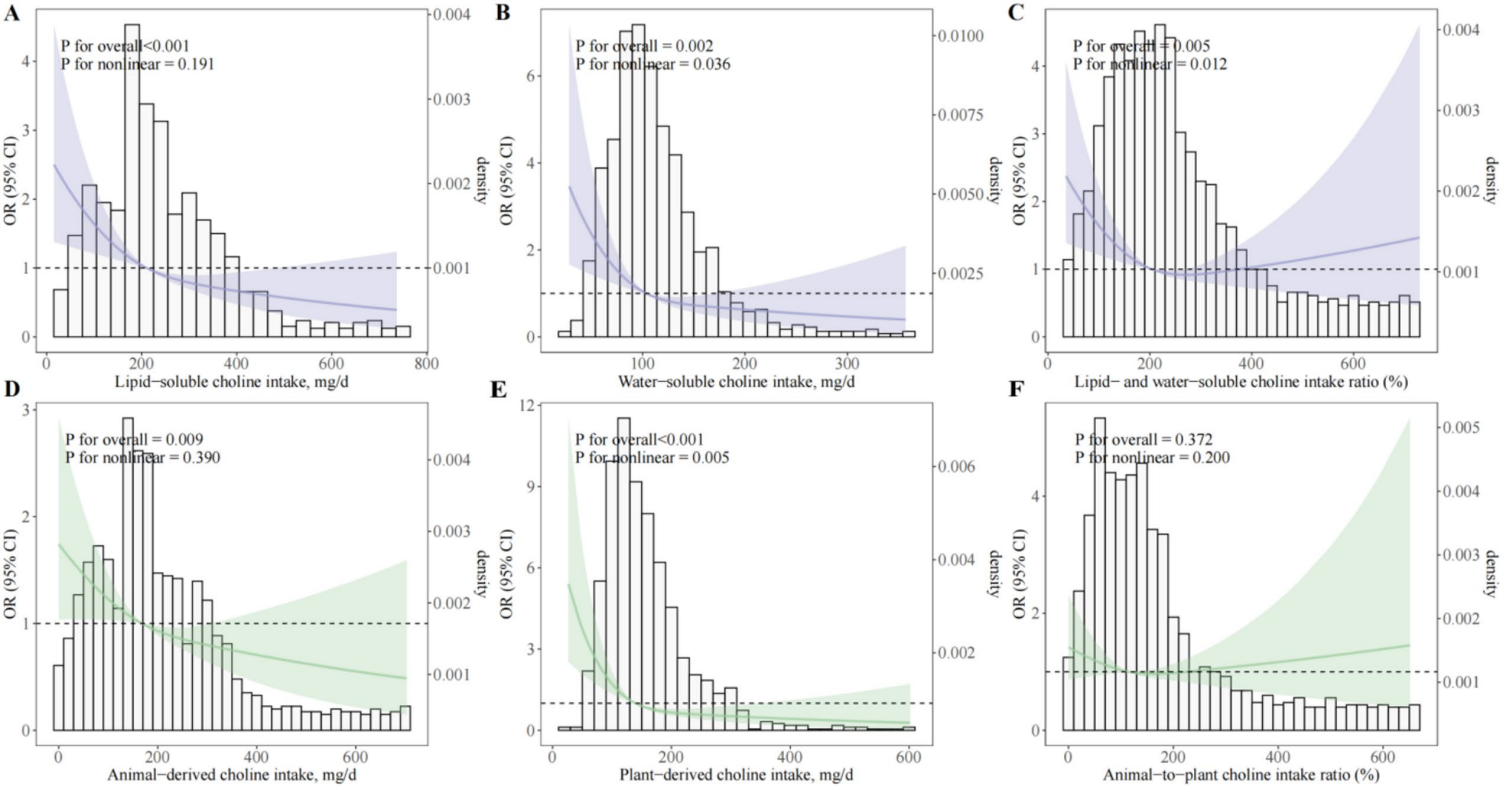
Dose–response associations between choline subtypes and sources and odds of preeclampsia. **(A)** Lipid-soluble choline (sum of PtdCho and SM); **(B)** Water-soluble choline (sum of free choline, GPCho, and PCho); **(C)** Ratio of lipid- to water-soluble choline intake; **(D)** Animal-derived choline intake; **(E)** Plant-derived choline intake; **(F)** Animal-to-plant choline intake ratio. OR, odds ratio; CI, confidence interval; BMI, body mass index; GDM, gestational diabetes mellitus; PCho, phosphocholine; GPCho, glycerophosphocholine; PtdCho, phosphatidylcholine; SM, sphingomyelin. Restricted cubic spline regression models depicting multivariable-adjusted dose–response relationships between various forms and dietary sources of choline intake and the odds of PE among pregnant women. All models were adjusted for maternal age (years), gestational age at survey (weeks), pre-pregnancy BMI (kg/m^2^), monthly household income, educational attainment, physical activity (MET-hours/day), employment status, smoking status, alcohol consumption, sleep quality, daily energy intake (kcal/day), season of dietary assessment, parity, GDM, menstrual regularity, family history of hypertension, and supplement use (folic acid and multivitamins).

Analysis of choline subcategories showed that both lipid- and water-soluble choline intakes were independently and inversely associated with the odds of PE (Figure 3; Supplementary Table 3). In fully adjusted models, OR_Q4 versus Q1_ was 0.33 (95% CI, 0.22–0.48; *P*_trend_ < 0.001) for lipid-soluble and 0.37 (95% CI, 0.25–0.54; *P*_trend_ < 0.001) for water-soluble choline. Dose–response analyses confirmed a significant linear association for lipid-soluble choline (*P*_overall_ < 0.001; *P*_nonlinear_ = 0.191; Figure 3A) and a modest nonlinearity for water-soluble choline (*P*_overall_ = 0.002; *P*_nonlinear_ = 0.036; Figure 3B). For the lipid-to-water choline intake ratio, an L-shaped association was observed, with the greatest odds reduction at moderate ratios (OR_Q3 versus Q1_ = 0.56; 95% CI, 0.39–0.82), but no further reduction at the highest quartile (OR_Q4 versus Q1_ = 0.72; 95% CI, 0.51–1.03; *P*_trend_ = 0.02; Figure 3C).

Source-specific analyses indicated that both animal-derived and plant-derived choline were inversely associated with PE odds (OR_Q4 versus Q1_ = 0.43, 95% CI, 0.30–0.63 and 0.31, 95% CI, 0.21–0.46, respectively; both *P*_trend_ < 0.001; Supplementary Table 4), with no significant association for the animal-to-plant choline intake ratio (OR_Q4 versus Q1_ = 0.96; 95% CI, 0.67–1.37; *P*_trend_ = 0.98). Egg consumption—the predominant dietary source of choline—was also inversely associated with PE odds (OR_Q4 versus Q1_ = 0.48; 95% CI, 0.32–0.72; *P*_trend_ < 0.001), with each 25 g/day increment corresponding to an OR of 0.89 (95% CI, 0.82–0.98; *p* = 0.01; Supplementary Table 5). Dose–response modeling indicated a consistent linear inverse association across the observed range of egg intake (*P*_overall_ = 0.005; *P*_nonlinear_ = 0.841; Supplementary Figure 3).

### 3.3 Stratified and sensitivity analyses

**Figure 4:**
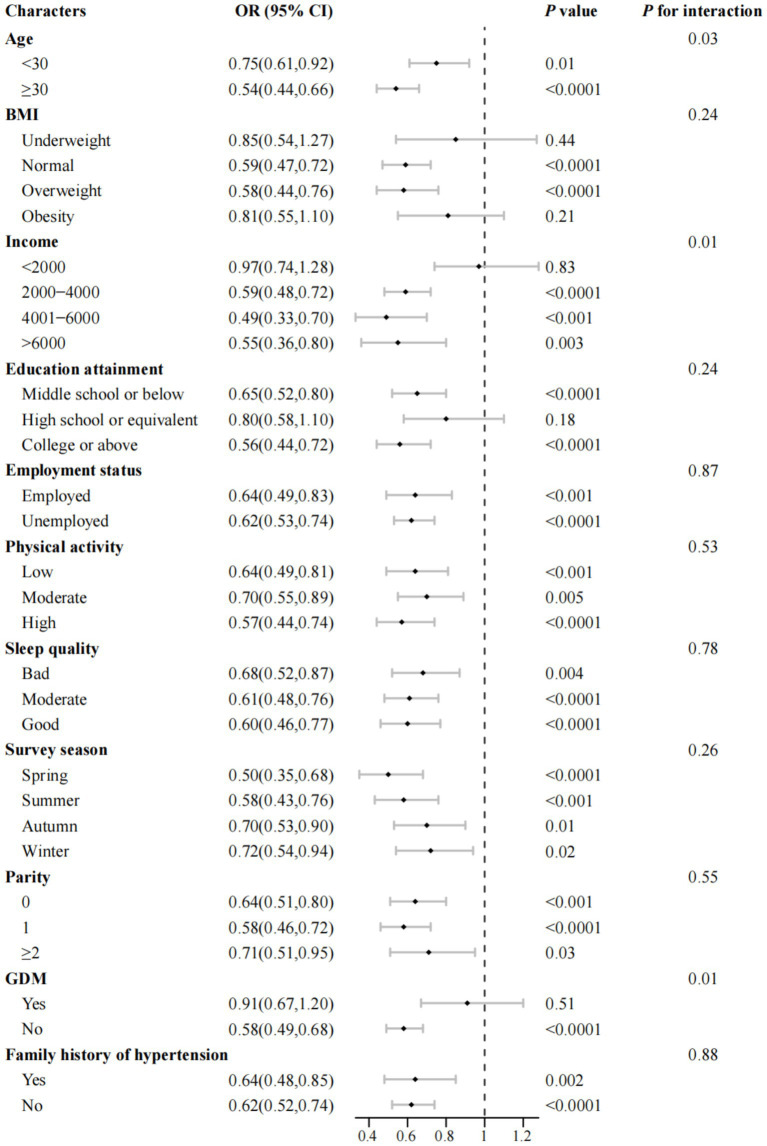
Association between total dietary choline intake and odds of preeclampsia across subgroups of maternal characteristics (*n* = 982). OR, odds ratio; CI, confidence interval; BMI, body mass index; GDM, gestational diabetes mellitus. Multivariable-adjusted ORs and corresponding 95% CIs for preeclampsia are presented per one standard deviation increase in total dietary choline intake, stratified by key maternal and lifestyle characteristics. All models were adjusted for maternal age group, gestational age at survey (weeks), pre-pregnancy BMI group, monthly household income, educational attainment, physical activity (MET-hours/day), employment status, smoking status, alcohol consumption, sleep quality, daily energy intake (kcal/day), season of dietary assessment, parity, GDM, menstrual regularity, family history of hypertension, and supplement use (folic acid and multivitamins), with the stratification variable excluded from each respective model. Point estimates (squares) indicate adjusted ORs, with error bars representing 95% CIs. *p* values for interaction were derived from likelihood ratio tests and are presented without adjustment for multiple comparisons.

We conducted stratified analyses and found that the inverse association between total dietary choline intake and the odds of PE was largely consistent across sociodemographic, lifestyle, reproductive, and clinical subgroups (Figure 4; Supplementary Table 6). For each 1-SD increment in choline intake, significant reductions in odds were observed for both age groups (<30 years: OR = 0.75; 95% CI, 0.61–0.92; ≥30 years: OR = 0.54; 95% CI, 0.44–0.66). Notably, the associations appeared stronger among women aged ≥30 years, those with higher income, and those without GDM. Significant interactions were detected in Figure 4 for age, income, and GDM (all *P*_interactio*n*_ < 0.05), but not for other variables (all *P*_interactio*n*_ > 0.05); no significant effect modification was found in Supplementary Table 6. Similarly, egg consumption showed consistent inverse associations across subgroups (Supplementary Figure 4), with significant interactions for age, income, and GDM, but not for other factors (all *P*_interactio*n*_ < 0.05).

Multiple sensitivity analyses consistently confirmed the robustness of our primary findings. When analyses were restricted to participants with complete data (excluding imputed values), higher total dietary choline intake remained strongly and inversely associated with the odds of PE (OR_Q4 versus Q1_ = 0.46, 95% CI: 0.28–0.76; *P*_trend_ = 0.001), and consistent inverse associations were observed for all major choline subtypes (Supplementary Table 7). Moreover, additional adjustment for psychological distress (anxiety and depression scores) did not meaningfully change the results (OR_Q4 versus Q1_ = 0.39, 95% CI: 0.24–0.64; *P*_trend_ < 0.001; Supplementary Table 8). Similarly, excluding participants with gestational diabetes yielded comparable associations (OR_Q4 versus Q1_ = 0.46, 95% CI: 0.28–0.78; *P*_trend_ < 0.001; Supplementary Table 9). In addition, analyses conducted without adjusting for total energy intake, as well as those using residual energy-adjusted choline intake, provided similar findings, further supporting the stability of our results (OR_Q4 versus Q1_ = 0.37–0.42, all *P*_trend_ < 0.001; Supplementary Tables 10, 11).

Furthermore, dose–response analyses based on restricted cubic spline models showed a significant linear inverse association between total choline intake and the odds of PE (*P*_overall_ < 0.001; *P*_nonlinear_ = 0.795), whereas no significant association was detected for betaine intake (*P*_overall_ = 0.200; *P*_nonlinear_ = 0.076; Supplementary Figure 5). Notably, similar linear trends were consistently observed for all choline subtypes. Collectively, these comprehensive sensitivity analyses reinforce the reliability and robustness of the observed inverse association between dietary choline intake and the odds of PE, regardless of analytic approach or potential confounding factors.

## 4 Discussion

In this matched case–control study of pregnant women in China, higher habitual intake of total dietary choline was associated with lower odds of preeclampsia (PE), after multivariable adjustment. Inverse associations were observed across major choline subtypes—including lipid-soluble phosphatidylcholine (PtdCho) and sphingomyelin (SM), as well as water-soluble free choline, glycerophosphocholine (GPCho), and phosphocholine (PCho)—and for both animal- and plant-derived sources. To our knowledge, within an Asian population, this is among the first studies to jointly assess amount, subtype distribution, dietary sources, and intake ratios of choline—including key contributors such as eggs—in relation to PE. These findings extend current evidence and suggest that adequate and diverse choline intake in early pregnancy may be relevant to PE prevention.

Choline is an essential nutrient with critical roles in membrane integrity, neurotransmitter synthesis, and one-carbon metabolism (13). In non-pregnant populations, higher intake has been linked to reduced cardiovascular, cognitive, and hepatic risks, including the Framingham Heart Study, which reported lower dementia and Alzheimer’s disease risk with moderate intake (31), and National Health and Nutrition Examination Survey analyses showing inverse associations with cardiovascular disease and stroke (32). Pregnancy-specific evidence is limited: in a prospective Iranian cohort, higher choline intake was associated with lower hypertension risk in women (33), whereas a large U.S. birth cohort found no association with gestational diabetes (34). Few studies have examined PE directly, and most have focused on total choline without differentiating chemical subtypes, sources, or intake ratios—particularly in Asian populations.

Our case–control study bridged these gaps by investigating lipid- and water-soluble subtypes, subtype proportions, and source-based intakes. Both subtype groups were inversely and individually associated with PE, and dose–response relationships revealed approximately linear patterns for lipid-soluble types and modest nonlinearity for water-soluble types (*P*_nonlinear_ = 0.036). The balance between lipid:water was L-shaped, and the relative maximum reduction was observed for a moderate balance. Our findings are consistent with prior evidence that plant-based, water-soluble choline is co-consumed with phytochemicals and unsaturated fats that may confer anti-inflammatory benefits (35, 36), whereas egg-derived PtdCho shows higher bioavailability than some synthetic forms (37) and may have higher membrane structure and methyl-donor economy impacts (38).

In our source-specific models, both plant- and animal-derived choline were inversely associated with PE risk, yet animal-to-plant ratio did not achieve significance in terms of outcome. This trend is consistent with nearly equivalent contribution from both sources, potentially easing complementing nutrient patterns. A South African birth cohort concluded that dairy- and egg-derived choline contributing >40% total choline had an associated 32% lower risk of PE (39). In another instance, among Norwegian stable angina patients, animal-derived choline provided 55%, whereas plant-derived provided 45%, to total choline, and specific molecular forms had distinct metabolic fates (40). Although prior evidence comes from non-pregnant populations, those findings are directionally consistent with our results and with mechanistic data indicating that adequate, source-diverse choline intake may influence cardiometabolic pathways. In our data, this intake was associated with lower odds of PE. Eggs, the leading contributor in our study population, had a dose–response association: 25 g/day increment (approximately one-half an egg) had 11% lower odds of PE. In agreement with previous studies, daily egg consumption significantly increases plasma choline and may promote fetal neuromaturation (41, 42), and experimental evidence further suggests that egg-derived choline attenuates PE-like features via α7-nAChR-mediated inhibition of NF-κB (43). Additionally, a recent Mendelian randomization analysis shows an inverse association between genetically proxied circulating choline levels and the risk of PE (20), providing genetic evidence consistent with a potential protective effect.

A variety of biologically reasonable mechanisms can support the observed inverse association between dietary choline intake and PE risk. First, epigenetic regulation via one-carbon metabolism. Choline, through betaine, supplies methyl groups for homocysteine remethylation and S-adenosylmethionine production (44, 45). Experimental studies show that maternal choline supplementation can reverse placental DNA hypomethylation, restore angiogenic gene expression, and modulate imprinted genes critical for placentation (46, 47). Furthermore, human evidence also links elevated choline intake to desirable placental methylation patterns (48). The superior bioavailability of egg-based PtdCho could increase methylation capacity, and plant-based, water-soluble sources could supply phytochemicals modifying methyltransferase activity. Second, antioxidant, anti-inflammatory, and vascular pathways. Choline participates in one-carbon metabolism and membrane phospholipid synthesis, processes that can influence redox balance and endothelial function (43). For example, PtdCho–enriched HDL supports endothelial homeostasis, whereas endothelial dysfunction in PE is linked to oxidative and inflammatory stress (49). Experimental data indicate that higher dietary choline can modulate placental angiogenic signaling—including vascular endothelial growth factor (VEGF)—and attenuate apoptotic and inflammatory responses, while choline deficiency shows opposite effects (46, 50). From a dietary perspective, plant-based, water-soluble choline may confer anti-inflammatory benefits, whereas egg-derived PtdCho supports membrane phospholipid integrity and lipoprotein structure (42, 51). Taken together with observational and genetic evidence, these findings support the biological plausibility that adequate, source-diverse choline intake may favorably influence epigenetic and vascular pathways relevant to PE.

Our study has a number of strengths. Most notably, our carefully matched case–control study design and validated food frequency questionnaire (FFQ) allowed careful measurement of total, subtype, ratio-based, and source-based choline intakes. Extensive use of sensitivity and subgroup analysis further strengthens the validity and generalizability of these findings. However, a number of limitations should be carefully considered. First, the study is necessarily of a case–control design, and causal inference is ruled out; reverse causation cannot be excluded. Second, FFQ-based measurement of exposure is susceptible to recall and misclassification error, and use of food-composition values, in part drawn from non-Chinese sources, may contribute additional measurement uncertainty. Third, although vigorous adjustment was made for a comprehensive array of known and suspected confounders, residual confounding by undiagnosed or imprecisely measured variables (e.g., genetic susceptibility, other dietary exposures, composition of gut microbiota) may be a factor. Fourth, the moderately sized sample size and associated sample size limitations may reduce statistical power to detect modest relationships or subtle interaction, and generalizability to other groups with different dietary patterns or racial/ethnic backgrounds may be attenuated. As a partial offset, during data collection, strict quality-assurance procedures were undertaken, and a complete range of sensitivity and subgroup analyses was undertaken, and these yielded results congruent and broadly supportive across all measures examined. Prospective studies, repeated dietary measures, and biomarker standardization will be critical future investigations to confirm these relationships. Overall, despite these challenges, our rigorous and detailed examination of choline subcategories and dietary sources offers strong and new mechanistic insight into dietary choline optimization as a preventative strategy for PE.

## 5 Conclusion

Higher total and subtype-specific dietary intakes of choline, both from animals and vegetables, were inversely associated with odds of PE in pregnant Chinese women. These findings point to the potential relevance of appropriate and diversified choline nutrition early in pregnancy, although prospective and interventional studies are needed to define causality and optimal levels of intake.

## Data Availability

The raw data supporting the conclusions of this article will be made available by the authors, without undue reservation.
