# Supplementary material for: Association between dietary choline intake and odds of preeclampsia: a case–control study
Source: Front Nutr. 2025 Nov 4;12:1703117. doi: 10.3389/fnut.2025.1703117 (PMC12623378; doi:10.3389/fnut.2025.1703117)
Supplement: Supplementary file 1 [file Table_1.docx]

**Supplementary materials**

**Supplementary Figures**

Supplementary Fig. 1. Flowchart of participant selection in the case–control study

Supplementary Fig. 2. Spearman correlation matrix of individual dietary choline components

Supplementary Fig. 3. Dose–response association between egg consumption and odds of preeclampsia

Supplementary Fig. 4. Odds ratios and 95% confidence intervals for preeclampsia per 25-unit increase in egg consumption, stratified by participant characteristics (n = 982)

Supplementary Fig. 5. Dose–response associations of dietary choline and betaine intake with preeclampsia in sensitivity analysis using residual energy-adjusted intake

**Supplementary Tables**

Supplementary Table 1. Baseline characteristics of participants across quartiles of total dietary choline intake

Supplementary Table 2. Distribution of total dietary choline and betaine intake by food source

Supplementary Table 3. Associations of lipid-soluble choline, water-soluble choline, and their intake ratio with odds of preeclampsia

Supplementary Table 4. Associations of choline intake from specific food sources with odds of preeclampsia

Supplementary Table 5. Associations between egg consumption and odds of preeclampsia

Supplementary table 6. Stratified associations between total dietary choline intake and preeclampsia odds across key participant subgroups (n = 982)

Supplementary Table 7. Sensitivity analysis of the associations between total choline intake and preeclampsia after excluding imputed data

Supplementary Table 8. Sensitivity analysis adjusting for psychological distress (anxiety and depression scores) in the association between choline intake and preeclampsia

Supplementary Table 9. Sensitivity analysis excluding participants diagnosed with gestational diabetes mellitus

Supplementary Table 10. Sensitivity analysis of associations between choline intake and preeclampsia, irrespective of total energy intake

Supplementary Table 11. Sensitivity analysis of associations between residual energy-adjusted choline intake and preeclampsia odds

**
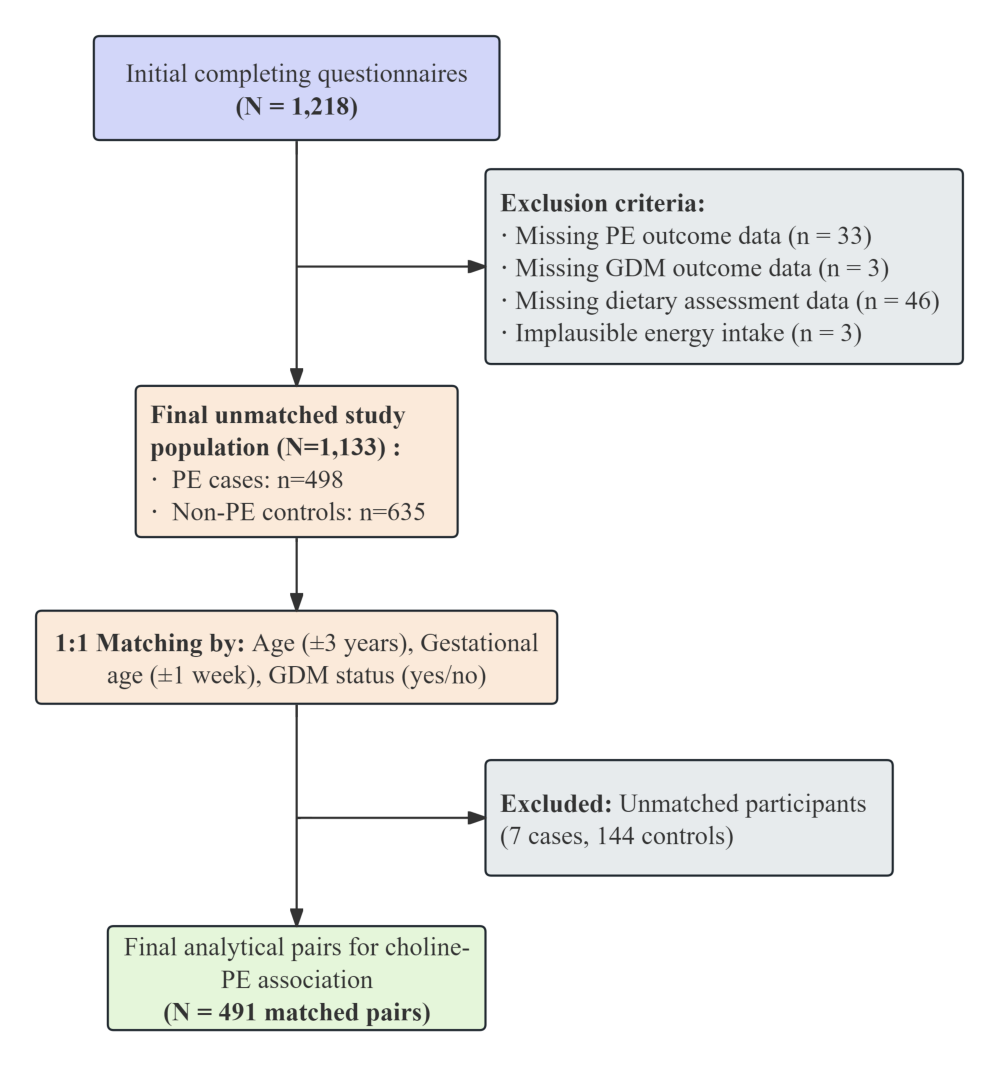
**

**Supplementary Fig. 1. Flowchart of participant selection in the case–control study**

**Abbreviations:** PE, preeclampsia; GDM, gestational diabetes mellitus.

This flowchart outlines the sequential exclusion criteria applied to derive the final analytic sample. Participants were excluded if they had multiple gestations, missing dietary or outcome data, or implausible energy intake, which was defined as >5,000 or ≤500 kcal/day.


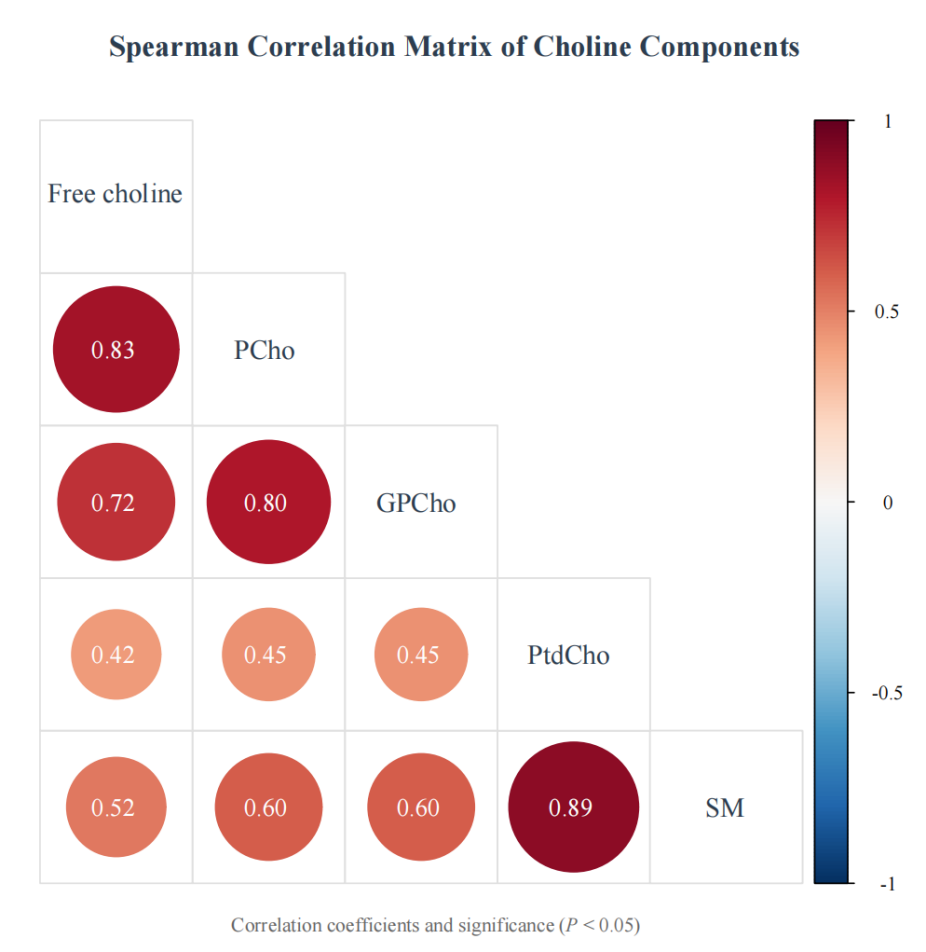


**Supplementary Fig. 2. Spearman correlation matrix of individual dietary choline components**

**Abbreviations:** PtdCho, phosphatidylcholine; SM, sphingomyelin; GPCho, glycerophosphocholine; PCho, phosphocholine.

This matrix displays Spearman correlation coefficients between individual choline components derived from dietary intake data. All correlations shown are statistically significant at P < 0.05. Higher positive correlations were observed among lipid-soluble components (PtdCho and SM) as well as between water-soluble components (Free choline, GPCho and PCho).


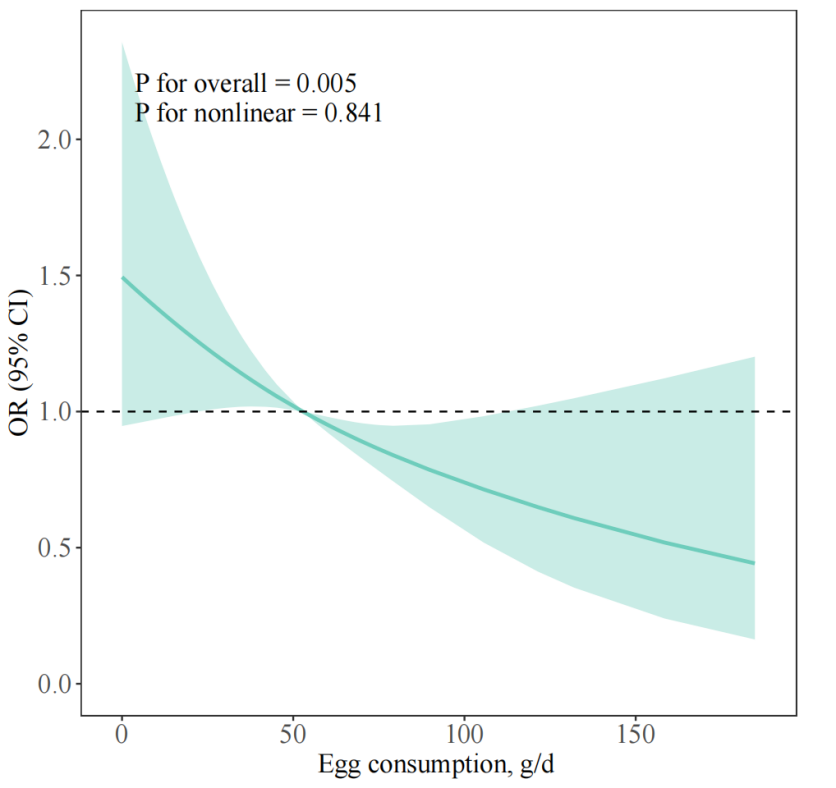


**Supplementary Fig. 3. Dose–response association between egg consumption and odds of preeclampsia**

Restricted cubic spline regression illustrating the multivariable-adjusted dose–response relationship between daily egg consumption (g/day) and odds of preeclampsia. All models were adjusted for maternal age (years), gestational age at survey (weeks), pre-pregnancy BMI (kg/m²), monthly household income, educational attainment, physical activity (MET-hours/day), employment status, smoking status, alcohol consumption, sleep quality, daily energy intake (kcal/day), season of dietary assessment, parity, GDM, menstrual regularity, family history of hypertension, and supplement use (folic acid and multivitamins).


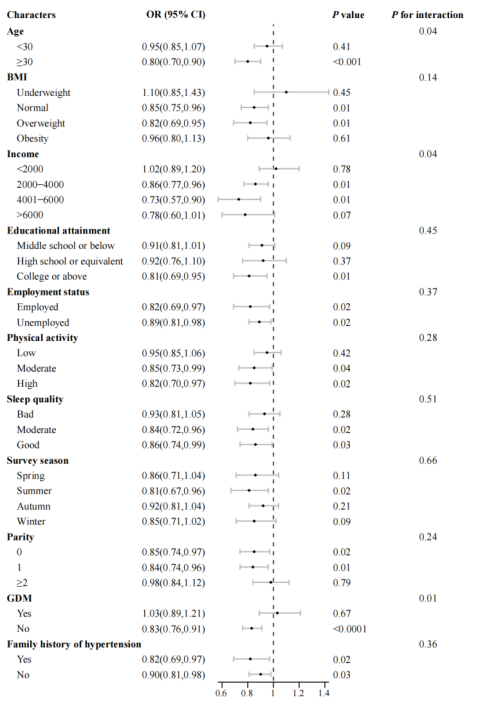


**Supplementary Fig. 4. Associations between egg consumption and odds of preeclampsia subgroups of maternal characteristics (n = 982)**

**Abbreviations:** OR, odds ratio; CI, confidence interval; BMI, body mass index; GDM, gestational diabetes mellitus.

Multivariable-adjusted odds ratios and 95% confidence intervals for preeclampsia are shown per 25 g/day increase in egg consumption, stratified by key maternal and lifestyle characteristics. All models were adjusted for maternal age group, gestational age at survey (weeks), pre-pregnancy BMI group, monthly household income, educational attainment, physical activity (MET-hours/day), employment status, smoking status, alcohol consumption, sleep quality, daily energy intake (kcal/day), season of dietary assessment, parity, GDM, menstrual regularity, family history of hypertension, and supplement use (folic acid and multivitamins), , with the stratification variable excluded from each respective model. Point estimates (squares) indicate adjusted ORs, with error bars representing 95% CIs. *P* values for interaction were derived from likelihood ratio tests and are presented without adjustment for multiple comparisons.


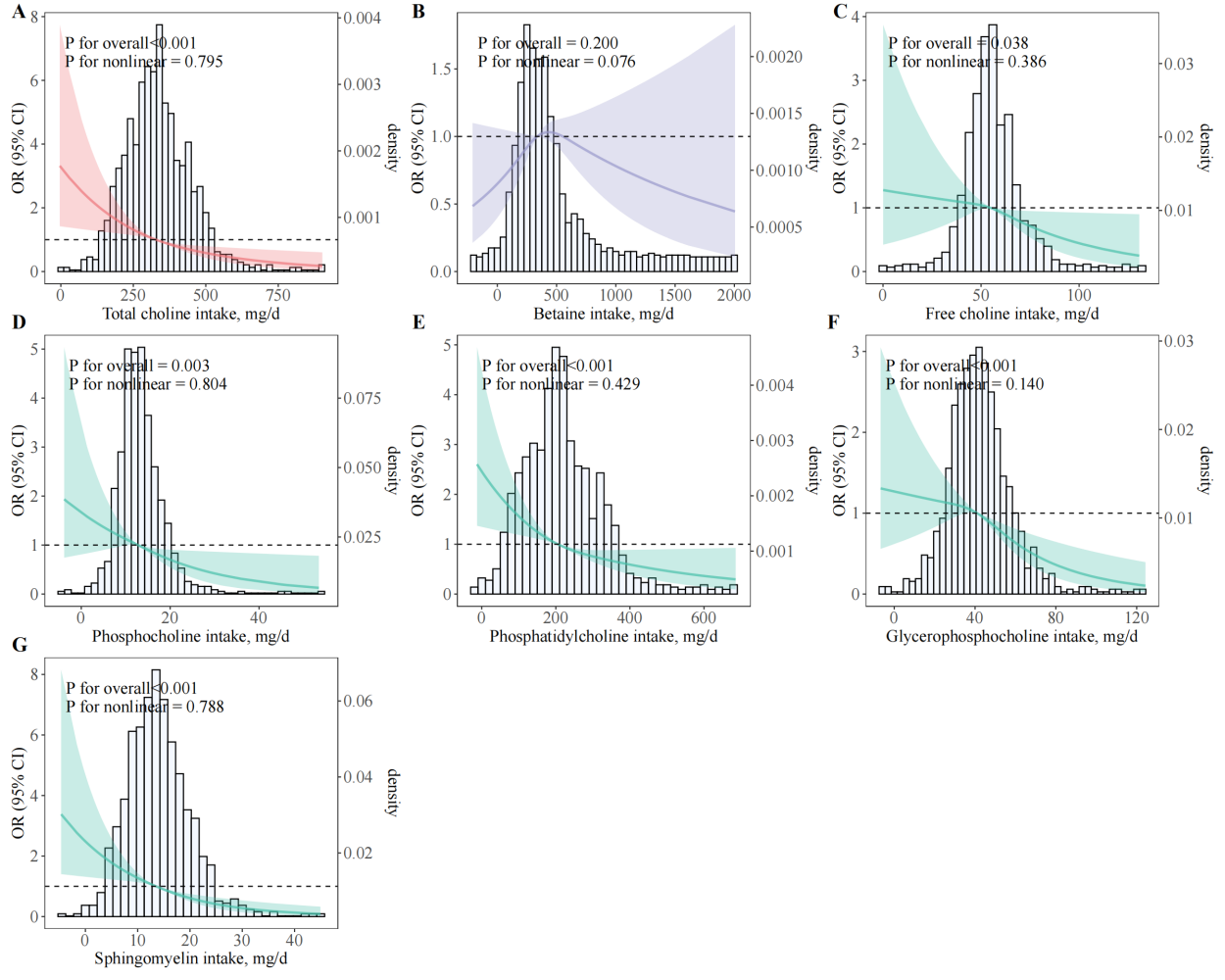


**Supplementary Fig. 5. Dose–response associations of dietary choline and betaine intake with preeclampsia in sensitivity analysis using residual energy-adjusted intake**

**(A)**Total choline; **(B)** Betaine; **(C)** Free choline; **(D)** PCho; **(E)** PtdCho; **(F)** GPCho; **(G)** SM;

**Abbreviations:** OR, odds ratio; CI, confidence interval; BMI, body mass index; GDM, gestational diabetes mellitus; PCho, phosphocholine; GPCho, glycerophosphocholine; PtdCho, phosphatidylcholine; SM, sphingomyelin.

Restricted cubic spline regression models illustrating multivariable-adjusted dose–response relationships between various forms of dietary choline and betaine intake and the odds of PE among pregnant women. All models were adjusted for maternal age (years), gestational age at survey (weeks), pre-pregnancy BMI (kg/m²), monthly household income, educational attainment, physical activity (MET-hours/day), employment status, smoking status, alcohol consumption, sleep quality, daily energy intake (kcal/day), season of dietary assessment, parity, GDM, menstrual regularity, family history of hypertension, and supplement use (folic acid and multivitamins).

**Supplementary Table 1. Baseline characteristics of participants stratified by preeclampsia status**

| **Characteristics** | **Total (*n* = 982)** | **Controls (n = 491)** | **Cases (n = 491)** | ***P* value** |
| --- | --- | --- | --- | --- |
| **Maternal age, years** | 31.05 ± 5.02 | 31.10 ± 4.78 | 31.00 ± 5.26 | 0.74 |
| **Gestational age, weeks** | 34.19 ± 2.84 | 34.52 ± 2.68 | 33.87 ± 2.96 | <0.001 |
| **Pre-pregnancy BMI, kg/m^2^** | 23.11 ± 3.63 | 22.54 ± 3.36 | 23.67 ± 3.79 | <0.001 |
| **Physical activity (MET- hours/d)** | 26.87 ± 4.31 | 26.83 ± 4.71 | 26.91 ± 3.87 | 0.77 |
| **Daily energy intake (kcal/day)** | 1920.00 ± 547.69 | 1997.97 ± 564.28 | 1842.04 ± 519.55 | <0.001 |
| **Anxiety score** | 38.25 ± 6.97 | 37.22 ± 6.45 | 39.28 ± 7.32 | <0.001 |
| **Depression score** | 39.62 ± 8.95 | 38.17 ± 8.61 | 41.08 ± 9.07 | <0.001 |
| **Income (Yuan/month)** |  |  |  | 0.21 |
| ≤2,000 | 128(13.03) | 55(11.20) | 73(14.87) |  |
| 2,001–4,000 | 523(53.26) | 260(52.95) | 263(53.56) |  |
| 4,001–6,000 | 180(18.33) | 92(18.74) | 88(17.92) |  |
| >6,000 | 151(15.38) | 84(17.11) | 67(13.65) |  |
| **Educational attainment, %** |  |  |  | <0.001 |
| Middle school or below | 405(41.24) | 175(35.64) | 230(46.84) |  |
| High school or equivalent | 184(18.74) | 92(18.74) | 92(18.74) |  |
| College or above | 393(40.02) | 224(45.62) | 169(34.42) |  |
| **Employment status, %** |  |  |  | 0.09 |
| Employed | 332(33.81) | 179(36.46) | 153(31.16) |  |
| Unemployed | 650(66.19) | 312(63.54) | 338(68.84) |  |
| **Smoking status, %** |  |  |  | 0.37 |
| Ever | 149(15.17) | 69(14.05) | 80(16.29) |  |
| Never | 833(84.83) | 422(85.95) | 411(83.71) |  |
| **Drinking status, %** |  |  |  | 0.50 |
| Ever | 20( 2.04) | 8( 1.63) | 12( 2.44) |  |
| Never | 962(97.96) | 483(98.37) | 479(97.56) |  |
| **Sleep quality, %** |  |  |  | <0.01 |
| Poor | 264(26.88) | 111(22.61) | 153(31.16) |  |
| Moderate | 396(40.33) | 213(43.38) | 183(37.27) |  |
| Good | 322(32.79) | 167(34.01) | 155(31.57) |  |
| **Survey season, %** |  |  |  | 0.04 |
| Spring | 267(27.19) | 153(31.16) | 114(23.22) |  |
| Summer | 211(21.49) | 102(20.77) | 109(22.20) |  |
| Autumn | 275(28.00) | 126(25.66) | 149(30.35) |  |
| Winter | 229(23.32) | 110(22.40) | 119(24.24) |  |
| **Parity, %** |  |  |  | <0.001 |
| 0 | 365(37.17) | 154(31.36) | 211(42.97) |  |
| 1 | 435(44.30) | 237(48.27) | 198(40.33) |  |
| ≥2 | 182(18.53) | 100(20.37) | 82(16.70) |  |
| **GDM, %** | 136(13.85) | 72(14.66) | 64(13.03) | 0.52 |
| **Menstrual regularity, %** | 909(92.57) | 466(94.91) | 443(90.22) | <0.01 |
| **Family history of hypertension, %** | 285(29.02) | 93(18.94) | 192(39.10) | <0.001 |
| **Dietary intake** |  |  |  |  |
| Egg (g/day) | 54.65 ± 34.62 | 59.85 ± 35.06 | 49.45 ± 33.40 | <0.001 |
| Vegetable (g/day) | 355.83 ± 172.79 | 378.54 ± 180.70 | 333.12 ± 161.52 | <0.001 |
| Fruit (g/day) | 363.84 ± 275.23 | 399.08 ± 302.64 | 328.60 ± 239.95 | <0.001 |
| Protein (g/day) | 66.32 ± 23.00 | 71.35 ± 23.74 | 61.29 ± 21.08 | <0.001 |
| Fat (g/day) | 74.77 ± 25.72 | 77.13 ± 24.99 | 72.41 ± 26.25 | <0.01 |
| Carbohydrate (g/day) | 252.40 ± 82.35 | 262.10 ± 88.11 | 242.70 ± 75.01 | <0.001 |
| Total choline (mg/day) | 335.84 ± 144.82 | 365.98 ± 140.07 | 305.70 ± 143.34 | <0.001 |
| Betaine (mg/day) | 372.16 ± 231.62 | 381.89 ± 241.26 | 362.42 ± 221.37 | 0.19 |
| Lipid-soluble choline (mg/day) | 226.02 ± 120.02 | 247.60 ± 111.09 | 204.44 ± 124.76 | <0.001 |
| Water-soluble choline (mg/day) | 109.82 ± 45.18 | 118.38 ± 48.86 | 101.26 ± 39.41 | <0.001 |
| Animal−derived choline (mg/day) | 180.82 ± 115.17 | 196.54 ± 106.71 | 165.10 ± 121.13 | 4.32 |
| Plant−derived choline (mg/day) | 155.02 ± 67.52 | 169.44 ± 71.48 | 140.60 ± 60.00 | 6.85 |
| Free choline (mg/day) | 54.96 ± 21.07 | 58.36 ± 22.52 | 51.56 ± 18.95 | <0.001 |
| **Phosphocholine** (mg/day) | 13.08 ± 6.28 | 14.18 ± 6.66 | 11.98 ± 5.67 | <0.001 |
| **Glycerophosphocholine**  (mg/day) | 41.78 ± 21.15 | 45.84 ± 23.31 | 37.72 ± 17.86 | <0.001 |
| **Phosphatidylcholine** (mg/day) | 212.24 ± 113.75 | 232.21 ± 105.02 | 192.28 ± 118.66 | <0.001 |
| **Sphingomyelin** (mg/day) | 13.78 ± 6.95 | 15.39 ± 6.90 | 12.16 ± 6.63 | <0.001 |
| Folic acid supplement, % | 792(80.65) | 396(80.65) | 396(80.65) | 1.00 |
| Multivitamin supplement, % | 128(13.03) | 65(13.24) | 63(12.83) | 0.92 |

**Abbreviations:** BMI, body mass index; GDM, gestational diabetes mellitus;

Continuous variables are presented as mean±standard deviation (SD), and categorical variables are expressed as number (percentage). Paired t-tests were used to compare continuous variables, while paired chi-squared tests were applied for categorical variables.

Two-sided *P* values are presented without adjustment for multiple comparisons, with *P* values below 0.001 reported as <0.001.

**Supplementary Table 2. Distribution of choline and betaine intake from various food sources.**

| **Group** | **Total choline** | **Betaine** | **Free choline** | **PCho** | **GPCho** | **PtdCho** | **SM** |
| --- | --- | --- | --- | --- | --- | --- | --- |
| Whole Grains | 10.01 | 51.61 | 25.97 | 2.68 | 21.55 | 4.37 | 2.16 |
| Legumes | 2.49 | 0.02 | 4.22 | 4.02 | 0.9 | 2.37 | 0 |
| Vegetables | 11.08 | 45.07 | 29.63 | 44.23 | 7.56 | 6.37 | 14.79 |
| Potatoes | 0 | 0 | 0 | 0 | 0 | 0 | 0 |
| Fruits | 6.89 | 0.15 | 16.48 | 9.02 | 10.34 | 3.91 | 0.14 |
| Red meats | 12.85 | 0.36 | 3.62 | 4.61 | 19.04 | 13.77 | 20.87 |
| Poultry | 1.65 | 0.12 | 0.77 | 2.12 | 0.22 | 1.75 | 7.07 |
| Seafoods | 0.76 | 2.17 | 0.39 | 0.31 | 0.5 | 0.98 | 0.05 |
| Eggs | **42.48** | 0.09 | 0.64 | 2.6 | 0.81 | 63.09 | 43.72 |
| Dairy foods | 8.92 | 0.37 | 12.57 | 27.95 | 38.31 | 0.7 | 11.2 |
| Nuts | 2.83 | 0.03 | 5.72 | 2.45 | 0.55 | 2.68 | 0 |
| All sweeteners | 0 | 0 | 0 | 0 | 0.01 | 0 | 0 |
| Tea | 0 | 0 | 0 | 0 | 0 | 0 | 0 |
| Coffee | 0 | 0 | 0 | 0 | 0 | 0 | 0 |
| Oils | 0.03 | 0.01 | 0 | 0 | 0.21 | 0 | 0 |

**Note:** Values represent the mean percentage of total daily intake of each choline and betaine subtype attributable to individual food groups. Proportions were calculated as the average contribution of each food group to overall intake across all participants, with results rounded to two decimal places. Among all sources, eggs contributed the highest proportion of total choline intake, accounting for 42.48%.

**Abbreviations:** PCho, phosphocholine; GPCho, glycerophosphocholine; PtdCho, phosphatidylcholine; SM, sphingomyelin.

**Supplementary Table 3. Associations of lipid-soluble choline, water-soluble choline, and their intake ratio with odds of preeclampsia**

|  | Cases/controls | Intake (mg/day) / Intake ratio (%) | Model 1 | | Model 2 | | Model 3 | |
| --- | --- | --- | --- | --- | --- | --- | --- | --- |
|  |  |  | OR (95% CI) | *P* value | OR (95% CI) | *P* value | OR (95% CI) | *P* value |
| **Lipid-soluble choline** |  |  |  |  |  |  |  |  |
| Q1 | 229 / 123 | 86.13 (15.41-124.89) | 1(reference) |  | 1(reference) |  | 1(reference) |  |
| Q2 | 90 / 123 | 167.11 (124.90-189.56) | 0.39(0.28,0.56) | <0.001 | 0.43(0.30,0.61) | <0.001 | 0.45(0.31,0.65) | <0.001 |
| Q3 | 103 / 122 | 222.54 (189.57-261.93) | 0.45(0.32,0.64) | <0.001 | 0.48(0.34,0.68) | <0.001 | 0.51(0.36,0.73) | <0.001 |
| Q4 | 69 / 123 | 330.24 (>261.93) | 0.3(0.21,0.44) | <0.001 | 0.32(0.22,0.47) | <0.001 | 0.33(0.22,0.48) | <0.001 |
| P for trend |  |  |  | <0.001 |  | <0.001 |  | <0.001 |
| Per SD increment |  |  | 0.66(0.57,0.76) | <0.001 | 0.68(0.59,0.79) | <0.001 | 0.69(0.60,0.80) | <0.001 |
| **Water-soluble choline** |  |  |  |  |  |  |  |  |
| Q1 | 218 / 123 | 62.83 (28.35-75.44) | 1(reference) |  | 1(reference) |  | 1(reference) |  |
| Q2 | 91 / 123 | 84.6 (75.44-95.60) | 0.42(0.29,0.59) | <0.001 | 0.44(0.31,0.62) | <0.001 | 0.47(0.32,0.67) | <0.001 |
| Q3 | 109 / 122 | 108.78 (95.60-122.94) | 0.5(0.36,0.71) | <0.001 | 0.53(0.38,0.75) | <0.001 | 0.55(0.39,0.79) | <0.001 |
| Q4 | 73 / 123 | 145.9 (>122.94) | 0.33(0.23,0.48) | <0.001 | 0.36(0.25,0.52) | <0.001 | 0.37(0.25,0.54) | <0.001 |
| P for trend |  |  |  | <0.001 |  | <0.001 |  | <0.001 |
| Per SD increment |  |  | 0.65(0.56,0.75) | <0.001 | 0.67(0.58,0.78) | <0.001 | 0.68(0.58,0.79) | <0.001 |
| **Lipid-water choline ratio** |  |  |  |  |  |  |  |  |
| Q1 | 166 / 123 | 101.37 (36.27-130.73) | 1(reference) |  | 1(reference) |  | 1(reference) |  |
| Q2 | 122 / 123 | 160 (130.73-193.49) | 0.73(0.52,1.03) | 0.08 | 0.77(0.54,1.09) | 0.14 | 0.81(0.57,1.16) | 0.25 |
| Q3 | 86 / 122 | 223.32 (193.49-266.28) | 0.52(0.36,0.75) | <0.001 | 0.55(0.38,0.80) | 0.002 | 0.56(0.39,0.82) | 0.003 |
| Q4 | 117 / 123 | 327.61 (>266.28) | 0.7(0.50,0.99) | 0.05 | 0.71(0.50,1.00) | 0.05 | 0.72(0.51,1.03) | 0.07 |
| P for trend |  |  |  | 0.01 |  | 0.01 |  | 0.02 |
| Per SD increment |  |  | 0.93(0.82,1.06) | 0.28 | 0.93(0.81,1.06) | 0.25 | 0.92(0.81,1.04) | 0.19 |

**Abbreviation:** Q, quartile; BMI, body mass index; GDM, gestational diabetes mellitus; OR, odds ratio; CI, confidence interval.

Multivariable logistic regression models were used to estimate ORs and 95% CIs for the association between quartiles of lipid-soluble choline, water-soluble choline, and their intake ratio with odds of preeclampsia. The lowest quartile (Q1) served as the reference category. P for trend was calculated by assigning the median value of each quartile as a continuous variable in the model. Per SD increment estimates reflect the association per one standard deviation increase in the dietary intake values.

Model 1 was adjusted for maternal age (years), gestational age at survey (weeks), and pre-pregnancy BMI (kg/m²).

Model 2 was further adjusted for socioeconomic and lifestyle factors, including monthly household income, educational attainment, physical activity (MET-hours/day), employment status, smoking status, alcohol consumption, and sleep quality.

Model 3 was additionally adjusted for dietary and reproductive variables, including daily energy intake (kcal/day), season of dietary assessment, parity, GDM, menstrual regularity, family history of hypertension, and supplement use (folic acid and multivitamins).

Two-sided *P* values are presented without adjustment for multiple comparisons, with *P* values below 0.001 reported as <0.001.

**Supplementary Table 4. Associations of choline intake from specific food sources with odds of preeclampsia**

|  | Cases/controls | Intake (mg/day) /  Intake ratio (%) | Model 1 | | Model 2 | | Model 3 | |
| --- | --- | --- | --- | --- | --- | --- | --- | --- |
|  |  |  | OR (95% CI) | *P* value | OR (95% CI) | *P* value | OR (95% CI) | *P* value |
| **Animal−derived choline intake** |  |  |  |  |  |  |  |  |
| Q1 | 192 / 123 | 51.28 (0.00-84.83) | 1(reference) |  | 1(reference) |  | 1(reference) |  |
| Q2 | 115 / 123 | 130.68 (84.84-151.70) | 0.6(0.43,0.84) | 0.003 | 0.63(0.45,0.89) | 0.01 | 0.67(0.47,0.95) | 0.02 |
| Q3 | 108 / 122 | 175.49 (151.71-217.12) | 0.57(0.40,0.80) | 0.001 | 0.6(0.42,0.85) | 0.004 | 0.67(0.47,0.96) | 0.03 |
| Q4 | 76 / 123 | 284.48 (>217.12) | 0.4(0.27,0.57) | <0.001 | 0.42(0.29,0.61) | <0.001 | 0.43(0.30,0.63) | <0.001 |
| P for trend |  |  |  | <0.001 |  | <0.001 |  | <0.001 |
| Per SD increment |  |  | 0.74(0.64,0.85) | <0.001 | 0.76(0.66,0.87) | <0.001 | 0.77(0.67,0.89) | <0.001 |
| **Plant−derived choline intake** |  |  |  |  |  |  |  |  |
| Q1 | 225 / 123 | 87.01 (26.51-98.51) | 1(reference) |  | 1(reference) |  | 1(reference) |  |
| Q2 | 118 / 123 | 111.96 (98.51-125.93) | 0.52(0.38,0.73) | <0.001 | 0.54(0.39,0.76) | <0.001 | 0.55(0.39,0.78) | <0.001 |
| Q3 | 82 / 122 | 142.34 (125.94-167.79) | 0.37(0.26,0.52) | <0.001 | 0.38(0.26,0.54) | <0.001 | 0.39(0.27,0.57) | <0.001 |
| Q4 | 66 / 123 | 203.67 (>167.79) | 0.29(0.20,0.43) | <0.001 | 0.31(0.21,0.45) | <0.001 | 0.31(0.21,0.46) | <0.001 |
| P for trend |  |  |  | <0.001 |  | <0.001 |  | <0.001 |
| Per SD increment |  |  | 0.62(0.54,0.72) | <0.001 | 0.63(0.55,0.73) | <0.001 | 0.64(0.55,0.74) | <0.001 |
| **Animal−to−plant choline intake ratio** |  |  |  |  |  |  |  |  |
| Q1 | 150 / 123 | 39.7 (0.00-61.62) | 1(reference) |  | 1(reference) |  | 1(reference) |  |
| Q2 | 96 / 123 | 80.97 (61.62-114.45) | 0.64(0.45,0.92) | 0.01 | 0.65(0.45,0.94) | 0.02 | 0.68(0.47,0.99) | 0.04 |
| Q3 | 115 / 122 | 143.45 (114.46-171.07) | 0.77(0.55,1.10) | 0.15 | 0.77(0.54,1.10) | 0.15 | 0.82(0.57,1.18) | 0.29 |
| Q4 | 130 / 123 | 212 (>171.07) | 0.87(0.62,1.22) | 0.41 | 0.9(0.64,1.28) | 0.57 | 0.96(0.67,1.37) | 0.80 |
| P for trend |  |  |  | 0.58 |  | 0.72 |  | 0.98 |
| Per SD increment |  |  | 1.01(0.89,1.14) | 0.93 | 1.01(0.89,1.15) | 0.89 | 1.01(0.88,1.15) | 0.91 |

**Abbreviation:** Q, quartile; BMI, body mass index; GDM, gestational diabetes mellitus; OR, odds ratio; CI, confidence interval.
Multivariable logistic regression models were used to estimate ORs and 95% CIs for the associations between quartiles of choline intake from animal-based foods, plant-based foods, and the animal-to-plant choline intake ratio and the odds of preeclampsia. The lowest quartile (Q1) served as the reference category. P for trend was calculated by assigning the median value of each quartile as a continuous variable in the model. Per SD increment estimates reflect the association per one standard deviation increase in the dietary intake values.

Model 1 was adjusted for maternal age (years), gestational age at survey (weeks), and pre-pregnancy BMI (kg/m²).

Model 2 was further adjusted for socioeconomic and lifestyle factors, including monthly household income, educational attainment, physical activity (MET-hours/day), employment status, smoking status, alcohol consumption, and sleep quality.

Model 3 was additionally adjusted for dietary and reproductive variables, including daily energy intake (kcal/day), season of dietary assessment, parity, GDM, menstrual regularity, family history of hypertension, and supplement use (folic acid and multivitamins).

Two-sided *P* values are presented without adjustment for multiple comparisons, with *P* values below 0.001 reported as <0.001.

**Supplementary Table 5. Associations between egg consumption and odds of preeclampsia**

|  | Cases/controls | Intake (g/day) | Model 1 | | Model 2 | | Model 3 | |
| --- | --- | --- | --- | --- | --- | --- | --- | --- |
|  |  |  | OR (95% CI) | *P* value | OR (95% CI) | *P* value | OR (95% CI) | *P* value |
| **Egg consumption quartiles** |  |  |  |  |  |  |  |  |
| Q1 | 203 / 131 | 15.09 (0.00-22.63) | 1(reference) |  | 1(reference) |  | 1(reference) |  |
| Q2 | 152 / 183 | 52.8 (22.63-52.80) | 0.57(0.41,0.77) | <0.001 | 0.6(0.44,0.83) | 0.002 | 0.63(0.45,0.88) | 0.01 |
| Q3 | 60 / 66 | 79.2 (52.81-79.20) | 0.59(0.39,0.90) | 0.01 | 0.62(0.41,0.96) | 0.03 | 0.61(0.39,0.95) | 0.03 |
| Q4 | 76 / 111 | 105.6 (>79.20) | 0.46(0.32,0.67) | <0.0001 | 0.47(0.32,0.69) | <0.0001 | 0.48(0.32,0.72) | <0.001 |
| P for trend |  |  |  | <0.0001 |  | <0.001 |  | <0.001 |
| Per 25-unit increment |  |  | 0.89(0.82,0.96) | 0.004 | 0.89(0.82,0.96) | 0.004 | 0.89(0.82,0.98) | 0.01 |

**Abbreviation:** Q, quartile; BMI, body mass index; GDM, gestational diabetes mellitus; OR, odds ratio; CI, confidence interval.

Multivariable logistic regression models were used to estimate ORs and 95% CIs for the associations between quartiles of egg consumption and the odds of preeclampsia. The lowest quartile (Q1) served as the reference category. P for trend was calculated by assigning the median value of each quartile as a continuous variable in the model. Per 25 g/day increment estimates reflect the association per 25-gram increase in daily egg consumption.

Model 1 was adjusted for maternal age (years), gestational age at survey (weeks), and pre-pregnancy BMI (kg/m²).

Model 2 was further adjusted for socioeconomic and lifestyle factors, including monthly household income, educational attainment, physical activity (MET-hours/day), employment status, smoking status, alcohol consumption, and sleep quality.

Model 3 was additionally adjusted for dietary and reproductive variables, including daily energy intake (kcal/day), season of dietary assessment, parity, GDM, menstrual regularity, family history of hypertension, and supplement use (folic acid and multivitamins).

Two-sided *P* values are presented without adjustment for multiple comparisons, with *P* values below 0.001 reported as <0.001.

**Supplementary table 6. Stratified associations between total dietary choline intake and preeclampsia odds across key participant subgroups (n = 982)**

| **Characters** | **Q1** | **Q2** | ***P*** | **Q3** | ***P*** | **Q4** | ***P*** | ***P* for trend** | ***P* for interaction** |
| --- | --- | --- | --- | --- | --- | --- | --- | --- | --- |
| **Age** |  |  |  |  |  |  |  |  | 0.08 |
| <30 | reference | 0.82(0.50,1.36) | 0.45 | 0.56(0.33,0.95) | 0.03 | 0.57(0.33,0.99) | 0.05 | 0.02 |  |
| ≥30 | reference | 0.54(0.34,0.83) | 0.01 | 0.30(0.18,0.49) | <0.001 | 0.22(0.13,0.37) | <0.001 | <0.001 |  |
| **BMI** |  |  |  |  |  |  |  |  | 0.87 |
| Underweight | reference | 1.11(0.29,4.19) | 0.88 | 0.45(0.09,1.94) | 0.31 | 0.77(0.21,2.73) | 0.69 | 0.49 |  |
| Normal | reference | 0.57(0.36,0.89) | 0.01 | 0.45(0.28,0.71) | <0.001 | 0.35(0.21,0.59) | <0.001 | <0.001 |  |
| Overweight | reference | 0.81(0.42,1.57) | 0.53 | 0.38(0.18,0.80) | 0.01 | 0.28(0.14,0.56) | <0.001 | <0.001 |  |
| Obesity | reference | 0.68(0.22,2.16) | 0.50 | 0.27(0.07,0.94) | 0.04 | 0.30(0.10,0.88) | 0.03 | 0.01 |  |
| **Income** |  |  |  |  |  |  |  |  | 0.27 |
| <2000 | reference | 0.59(0.24,1.48) | 0.26 | 0.59(0.19,1.83) | 0.36 | 0.76(0.29,1.98) | 0.57 | 0.45 |  |
| 2000-4000 | reference | 0.65(0.41,1.02) | 0.06 | 0.41(0.25,0.66) | <0.001 | 0.32(0.19,0.54) | <0.001 | <0.001 |  |
| 4001-6000 | reference | 0.57(0.26,1.24) | 0.16 | 0.18(0.07,0.43) | <0.001 | 0.30(0.12,0.70) | 0.01 | <0.001 |  |
| ＞6000 | reference | 0.86(0.36,2.03) | 0.73 | 0.77(0.31,1.90) | 0.57 | 0.24(0.08,0.66) | 0.01 | 0.01 |  |
| **Educational attainment** |  |  |  |  |  |  |  |  | 0.08 |
| Middle school or below | reference | 0.68(0.40,1.14) | 0.14 | 0.26(0.14,0.47) | <0.001 | 0.30(0.17,0.53) | <0.001 | <0.001 |  |
| High school or equivalent | reference | 0.54(0.25,1.14) | 0.11 | 1.11(0.47,2.65) | 0.82 | 0.53(0.23,1.17) | 0.12 | 0.28 |  |
| College or above | reference | 0.79(0.45,1.36) | 0.39 | 0.48(0.27,0.83) | 0.01 | 0.36(0.19,0.66) | 0.001 | <0.001 |  |
| **Employment status** |  |  |  |  |  |  |  |  | 0.44 |
| Employed | reference | 0.61(0.34,1.07) | 0.09 | 0.50(0.27,0.92) | 0.03 | 0.46(0.24,0.88) | 0.02 | 0.01 |  |
| Unemployed | reference | 0.69(0.46,1.04) | 0.08 | 0.36(0.23,0.56) | <0.001 | 0.29(0.19,0.46) | <0.001 | <0.001 |  |
| **Physical activity** |  |  |  |  |  |  |  |  | 0.18 |
| Low | reference | 0.83(0.46,1.50) | 0.54 | 0.46(0.25,0.85) | 0.01 | 0.25(0.12,0.48) | <0.001 | <0.001 |  |
| Moderate | reference | 0.63(0.36,1.11) | 0.11 | 0.35(0.18,0.66) | 0.001 | 0.63(0.33,1.22) | 0.17 | 0.02 |  |
| High | reference | 0.52(0.29,0.93) | 0.03 | 0.42(0.23,0.77) | 0.01 | 0.28(0.15,0.54) | <0.001 | <0.001 |  |
| **Sleep quality** |  |  |  |  |  |  |  |  | 0.88 |
| Bad | reference | 0.91(0.47,1.78) | 0.78 | 0.43(0.21,0.85) | 0.02 | 0.34(0.17,0.70) | 0.004 | <0.001 |  |
| Moderate | reference | 0.64(0.38,1.07) | 0.09 | 0.38(0.21,0.67) | <0.001 | 0.37(0.20,0.65) | <0.001 | <0.001 |  |
| Good | reference | 0.49(0.27,0.87) | 0.02 | 0.39(0.21,0.73) | 0.003 | 0.30(0.16,0.57) | <0.001 | <0.001 |  |
| **Survey season** |  |  |  |  |  |  |  |  | 0.97 |
| Spring | reference | 0.64(0.33,1.22) | 0.18 | 0.41(0.21,0.77) | 0.01 | 0.31(0.14,0.67) | 0.003 | <0.001 |  |
| Summer | reference | 0.64(0.30,1.33) | 0.23 | 0.29(0.12,0.66) | 0.004 | 0.25(0.11,0.53) | <0.001 | <0.001 |  |
| Autumn | reference | 0.65(0.34,1.23) | 0.18 | 0.44(0.22,0.86) | 0.02 | 0.32(0.15,0.64) | 0.001 | <0.001 |  |
| Winter | reference | 0.61(0.31,1.19) | 0.15 | 0.50(0.22,1.08) | 0.08 | 0.48(0.23,0.98) | 0.05 | 0.03 |  |
| **Parity** |  |  |  |  |  |  |  |  | 0.43 |
| 0 | reference | 0.78(0.44,1.37) | 0.39 | 0.59(0.33,1.05) | 0.07 | 0.38(0.21,0.69) | 0.002 | 0.001 |  |
| 1 | reference | 0.70(0.42,1.15) | 0.16 | 0.29(0.16,0.50) | <0.001 | 0.33(0.19,0.58) | <0.001 | <0.001 |  |
| ≥2 | reference | 0.39(0.18,0.82) | 0.01 | 0.38(0.15,0.91) | 0.03 | 0.29(0.12,0.67) | 0.01 | 0.002 |  |
| **GDM** |  |  |  |  |  |  |  |  | 0.77 |
| Yes | reference | 0.76(0.28,2.02) | 0.58 | 0.31(0.11,0.83) | 0.02 | 0.27(0.10,0.73) | 0.01 | 0.003 |  |
| No | reference | 0.63(0.44,0.89) | 0.01 | 0.42(0.28,0.62) | <0.001 | 0.35(0.24,0.53) | <0.001 | <0.001 |  |
| **Family history of**  **hypertension** |  |  |  |  |  |  |  |  | 0.16 |
| Yes | reference | 0.61(0.32,1.16) | 0.13 | 0.74(0.36,1.54) | 0.41 | 0.37(0.18,0.76) | 0.01 | 0.02 |  |
| No | reference | 0.64(0.43,0.95) | 0.03 | 0.32(0.20,0.49) | <0.001 | 0.33(0.21,0.51) | <0.001 | <0.001 |  |

**Supplementary table 6. Stratified associations between total dietary choline intake and preeclampsia odds across key participant subgroups (n = 982)**

**Abbreviations:** OR, odds ratio; CI, confidence interval; BMI, body mass index; GDM, gestational diabetes mellitus.

Multivariable-adjusted ORs and corresponding 95% CIs for preeclampsia are presented across quartiles of total dietary choline intake, stratified by key maternal and lifestyle characteristics. All models were adjusted for maternal age group, gestational age at survey (weeks), pre-pregnancy BMI group, monthly household income, educational attainment, physical activity (MET-hours/day), employment status, smoking status, alcohol consumption, sleep quality, daily energy intake (kcal/day), season of dietary assessment, parity, GDM, menstrual regularity, family history of hypertension, and supplement use (folic acid and multivitamins) , with the stratification variable excluded from each respective model. Point estimates (squares) indicate adjusted ORs, with error bars representing 95% CIs. P values for interaction were derived from likelihood ratio tests and are presented without adjustment for multiple comparisons.

Supplementary Table 7. Sensitivity analysis of the associations between total choline intake and preeclampsia after excluding imputed data

|  | Cases/controls | Model 1 | | Model 2 | | Model 3 | |
| --- | --- | --- | --- | --- | --- | --- | --- |
|  |  | OR (95% CI) | *P* value | OR (95% CI) | *P* value | OR (95% CI) | *P* value |
| **Total choline intake** | |  |  |  |  |  |  |
| Q1 | 206 / 123 | 1(reference) |  | 1(reference) |  | 1(reference) |  |
| Q2 | 133 / 123 | 0.73(0.52,1.02) | 0.07 | 0.82(0.57,1.16) | 0.26 | 0.92(0.63,1.36) | 0.69 |
| Q3 | 82 / 122 | 0.44(0.31,0.64) | <0.001 | 0.5(0.34,0.74) | <0.001 | 0.61(0.39,0.95) | 0.03 |
| Q4 | 70 / 123 | 0.37(0.25,0.54) | <0.001 | 0.39(0.26,0.57) | <0.001 | 0.46(0.28,0.76) | 0.002 |
| P for trend |  |  | <0.001 |  | <0.001 |  | 0.001 |
| Per SD increment |  | 0.65(0.56,0.75) | <0.001 | 0.67(0.58,0.77) | <0.001 | 0.72(0.59,0.87) | <0.001 |
| **Free choline** |  |  |  |  |  |  |  |
| Q1 | 192 / 123 | 1(reference) |  | 1(reference) |  | 1(reference) |  |
| Q2 | 110 / 123 | 0.57(0.40,0.80) | 0.001 | 0.58(0.41,0.83) | 0.003 | 0.61(0.43,0.88) | 0.01 |
| Q3 | 116 / 122 | 0.6(0.42,0.84) | 0.003 | 0.62(0.44,0.88) | 0.01 | 0.64(0.45,0.92) | 0.02 |
| Q4 | 73 / 123 | 0.38(0.27,0.55) | <0.001 | 0.4(0.27,0.58) | <0.001 | 0.38(0.26,0.57) | <0.001 |
| P for trend |  |  | <0.001 |  | <0.001 |  | <0.001 |
| Per SD increment |  | 0.71(0.62,0.82) | <0.001 | 0.72(0.63,0.83) | <0.001 | 0.72(0.62,0.83) | <0.001 |
| **Phosphocholine** |  |  |  |  |  |  |  |
| Q1 | 204 / 123 | 1(reference) |  | 1(reference) |  | 1(reference) |  |
| Q2 | 115 / 123 | 0.56(0.39,0.79) | <0.001 | 0.59(0.41,0.85) | 0.004 | 0.66(0.45,0.98) | 0.04 |
| Q3 | 103 / 122 | 0.55(0.39,0.79) | <0.001 | 0.57(0.40,0.83) | 0.003 | 0.62(0.41,0.95) | 0.03 |
| Q4 | 69 / 123 | 0.35(0.24,0.51) | <0.001 | 0.38(0.26,0.57) | <0.001 | 0.44(0.26,0.73) | 0.002 |
| P for trend |  |  | <0.001 |  | <0.001 |  | 0.002 |
| Per SD increment |  | 0.69(0.60,0.80) | <0.001 | 0.72(0.61,0.84) | <0.001 | 0.78(0.63,0.96) | 0.02 |
| **Glycerophosphocholine** | |  |  |  |  |  |  |
| Q1 | 200 / 123 | 1(reference) |  | 1(reference) |  | 1(reference) |  |
| Q2 | 122 / 123 | 0.61(0.43,0.86) | 0.005 | 0.62(0.43,0.89) | 0.01 | 0.65(0.44,0.97) | 0.03 |
| Q3 | 100 / 122 | 0.52(0.37,0.74) | <0.001 | 0.56(0.39,0.81) | 0.002 | 0.66(0.43,1.01) | 0.06 |
| Q4 | 69 / 123 | 0.37(0.26,0.55) | <0.001 | 0.41(0.28,0.61) | <0.001 | 0.54(0.32,0.92) | 0.02 |
| P for trend |  |  | <0.001 |  | <0.001 |  | 0.02 |
| Per SD increment |  | 0.66(0.57,0.77) | <0.001 | 0.68(0.58,0.80) | <0.001 | 0.75(0.60,0.94) | 0.01 |
| **Phosphatidylcholine** | |  |  |  |  |  |  |
| Q1 | 229 / 123 | 1(reference) |  | 1(reference) |  | 1(reference) |  |
| Q2 | 90 / 123 | 0.43(0.30,0.62) | <0.001 | 0.48(0.33,0.69) | <0.001 | 0.58(0.39,0.87) | 0.01 |
| Q3 | 101 / 122 | 0.47(0.33,0.67) | <0.001 | 0.53(0.37,0.77) | <0.001 | 0.61(0.41,0.92) | 0.02 |
| Q4 | 71 / 123 | 0.34(0.23,0.49) | <0.001 | 0.34(0.23,0.50) | <0.001 | 0.43(0.27,0.67) | <0.001 |
| P for trend |  |  | <0.001 |  | <0.001 |  | <0.001 |
| Per SD increment |  | 0.69(0.60,0.80) | <0.001 | 0.71(0.61,0.82) | <0.001 | 0.77(0.65,0.92) | 0.003 |
| **Sphingomyelin** |  |  |  |  |  |  |  |
| Q1 | 220 / 123 | 1(reference) |  | 1(reference) |  | 1(reference) |  |
| Q2 | 123 / 123 | 0.64(0.46,0.90) | 0.01 | 0.7(0.49,1.00) | 0.05 | 0.74(0.51,1.10) | 0.13 |
| Q3 | 92 / 122 | 0.44(0.31,0.62) | <0.001 | 0.52(0.35,0.75) | <0.001 | 0.63(0.41,0.95) | 0.03 |
| Q4 | 56 / 123 | 0.28(0.19,0.42) | <0.001 | 0.29(0.19,0.43) | <0.001 | 0.29(0.18,0.48) | <0.001 |
| P for trend |  |  | <0.001 |  | <0.001 |  | <0.001 |
| Per SD increment |  | 0.61(0.52,0.70) | <0.001 | 0.62(0.54,0.73) | <0.001 | 0.65(0.55,0.78) | <0.001 |
| **Betaine** |  |  |  |  |  |  |  |
| Q1 | 121 / 123 | 1(reference) |  | 1(reference) |  | 1(reference) |  |
| Q2 | 157 / 124 | 1.18(0.83,1.68) | 0.35 | 1.16(0.80,1.68) | 0.44 | 1.32(0.89,1.98) | 0.17 |
| Q3 | 108 / 121 | 0.87(0.60,1.26) | 0.45 | 0.81(0.55,1.19) | 0.27 | 1.07(0.70,1.64) | 0.76 |
| Q4 | 105 / 123 | 0.8(0.55,1.16) | 0.24 | 0.72(0.49,1.07) | 0.11 | 1.07(0.68,1.68) | 0.78 |
| P for trend |  |  | 0.1 |  | 0.03 |  | 0.96 |
| Per SD increment |  | 0.9(0.79,1.02) | 0.11 | 0.86(0.75,0.99) | 0.03 | 0.98(0.84,1.15) | 0.82 |

**Abbreviation:** Q, quartile; OR, odds ratio; CI, confidence interval; BMI, body mass index; GDM, gestational diabetes mellitus;

Multivariable logistic regression models were used to estimate ORs and 95% CIs for the association between quartiles of individual choline subtypes and betaine intake and the odds of preeclampsia. The lowest quartile (Q1) was used as the reference group. P for trend was derived by modeling the median value of each quartile as a continuous variable. Per standard deviation (SD) increment estimates represent the change in odds of preeclampsia per one SD increase in the intake values.

Model 1 was adjusted for maternal age (years), gestational age at survey (weeks), and pre-pregnancy BMI (kg/m²).

Model 2 was further adjusted for socioeconomic and lifestyle factors, including monthly household income, educational attainment, physical activity (MET-hours/day), employment status, smoking status, alcohol consumption, and sleep quality.

Model 3 was additionally adjusted for dietary and reproductive variables, including daily energy intake (kcal/day), season of dietary assessment, parity, GDM, menstrual regularity, family history of hypertension, and supplement use (folic acid and multivitamins).

Two-sided *P* values are presented without adjustment for multiple comparisons, with *P* values below 0.001 reported as <0.001.

Supplementary Table 8. Sensitivity analysis adjusting for psychological distress (anxiety and depression scores) in the association between choline intake and preeclampsia

|  | Cases/controls | Model 1 | | Model 2 | | Model 3 | | Model 4 | |
| --- | --- | --- | --- | --- | --- | --- | --- | --- | --- |
|  |  | OR (95% CI) | *P* value | OR (95% CI) | *P* value | OR (95% CI) | *P* value | OR (95% CI) | *P* value |
| **Total choline intake** | |  |  |  |  |  |  |  |  |
| Q1 | 206 / 123 | 1(reference) |  | 1(reference) |  | 1(reference) |  | 1(reference) |  |
| Q2 | 133 / 123 | 0.7(0.50,0.98) | 0.04 | 0.74(0.52,1.04) | 0.08 | 0.79(0.54,1.14) | 0.21 | 0.82(0.56,1.19) | 0.30 |
| Q3 | 82 / 122 | 0.43(0.30,0.62) | <0.001 | 0.46(0.32,0.67) | <0.001 | 0.54(0.36,0.82) | 0.004 | 0.54(0.35,0.83) | 0.005 |
| Q4 | 70 / 123 | 0.36(0.25,0.53) | <0.001 | 0.37(0.25,0.54) | <0.001 | 0.42(0.26,0.68) | <0.001 | 0.39(0.24,0.64) | <0.001 |
| P for trend |  |  | <0.001 |  | <0.001 |  | <0.001 |  | <0.001 |
| Per SD increment |  | 0.65(0.56,0.75) | <0.001 | 0.66(0.57,0.76) | <0.001 | 0.69(0.57,0.84) | <0.001 | 0.67(0.55,0.81) | <0.001 |
| **Free choline** |  |  |  |  |  |  |  |  |  |
| Q1 | 192 / 123 | 1(reference) |  | 1(reference) |  | 1(reference) |  | 1(reference) |  |
| Q2 | 110 / 123 | 0.57(0.41,0.81) | 0.001 | 0.59(0.42,0.84) | 0.003 | 0.62(0.43,0.88) | 0.01 | 0.62(0.42,0.91) | 0.02 |
| Q3 | 116 / 122 | 0.61(0.43,0.86) | 0.004 | 0.63(0.45,0.89) | 0.01 | 0.65(0.46,0.93) | 0.02 | 0.66(0.43,1.02) | 0.06 |
| Q4 | 73 / 123 | 0.38(0.26,0.55) | <0.001 | 0.39(0.27,0.57) | <0.001 | 0.39(0.27,0.57) | <0.001 | 0.42(0.23,0.76) | 0.004 |
| P for trend |  |  | <0.001 |  | <0.001 |  | <0.001 |  | 0.01 |
| Per SD increment |  | 0.71(0.62,0.81) | <0.001 | 0.72(0.62,0.83) | <0.001 | 0.72(0.62,0.83) | <0.001 | 0.75(0.59,0.96) | 0.02 |
| **Phosphocholine** |  |  |  |  |  |  |  |  |  |
| Q1 | 204 / 123 | 1(reference) |  | 1(reference) |  | 1(reference) |  | 1(reference) |  |
| Q2 | 115 / 123 | 0.59(0.42,0.83) | 0.003 | 0.64(0.45,0.91) | 0.01 | 0.69(0.48,1.01) | 0.06 | 0.73(0.50,1.07) | 0.10 |
| Q3 | 103 / 122 | 0.55(0.39,0.78) | <0.001 | 0.57(0.40,0.82) | 0.002 | 0.56(0.38,0.85) | 0.01 | 0.57(0.38,0.86) | 0.01 |
| Q4 | 69 / 123 | 0.36(0.25,0.52) | <0.001 | 0.39(0.26,0.57) | <0.001 | 0.45(0.27,0.73) | 0.001 | 0.46(0.28,0.75) | 0.002 |
| P for trend |  |  | <0.001 |  | <0.001 |  | <0.001 |  | <0.001 |
| Per SD increment |  | 0.69(0.60,0.80) | <0.001 | 0.71(0.61,0.83) | <0.001 | 0.75(0.61,0.92) | 0.01 | 0.75(0.61,0.92) | 0.01 |
| **Glycerophosphocholine** | |  |  |  |  |  |  |  |  |
| Q1 | 200 / 123 | 1(reference) |  | 1(reference) |  | 1(reference) |  | 1(reference) |  |
| Q2 | 122 / 123 | 0.64(0.46,0.90) | 0.01 | 0.69(0.49,0.98) | 0.04 | 0.71(0.49,1.04) | 0.08 | 0.74(0.50,1.08) | 0.12 |
| Q3 | 100 / 122 | 0.52(0.36,0.74) | <0.001 | 0.55(0.38,0.79) | 0.001 | 0.59(0.39,0.89) | 0.01 | 0.61(0.40,0.93) | 0.02 |
| Q4 | 69 / 123 | 0.38(0.26,0.56) | <0.001 | 0.4(0.27,0.59) | <0.001 | 0.5(0.30,0.83) | 0.01 | 0.5(0.30,0.83) | 0.01 |
| P for trend |  |  | <0.001 |  | <0.001 |  | 0.004 |  | 0.004 |
| Per SD increment |  | 0.66(0.57,0.77) | <0.001 | 0.68(0.58,0.79) | <0.001 | 0.72(0.58,0.90) | 0.003 | 0.72(0.58,0.90) | 0.004 |
| **Phosphatidylcholine** | |  |  |  |  |  |  |  |  |
| Q1 | 229 / 123 | 1(reference) |  | 1(reference) |  | 1(reference) |  | 1(reference) |  |
| Q2 | 90 / 123 | 0.42(0.30,0.60) | <0.001 | 0.45(0.31,0.64) | <0.001 | 0.5(0.34,0.73) | <0.001 | 0.53(0.36,0.78) | 0.001 |
| Q3 | 101 / 122 | 0.47(0.33,0.66) | <0.001 | 0.5(0.35,0.71) | <0.001 | 0.55(0.37,0.80) | 0.002 | 0.56(0.38,0.83) | 0.004 |
| Q4 | 71 / 123 | 0.33(0.23,0.48) | <0.001 | 0.33(0.23,0.49) | <0.001 | 0.4(0.26,0.61) | <0.001 | 0.38(0.25,0.59) | <0.001 |
| P for trend |  |  | <0.001 |  | <0.001 |  | <0.001 |  | <0.001 |
| Per SD increment |  | 0.69(0.59,0.79) | <0.001 | 0.7(0.60,0.80) | <0.001 | 0.76(0.64,0.89) | <0.001 | 0.73(0.62,0.87) | <0.001 |
| **Sphingomyelin** |  |  |  |  |  |  |  |  |  |
| Q1 | 220 / 123 | 1(reference) |  | 1(reference) |  | 1(reference) |  | 1(reference) |  |
| Q2 | 123 / 123 | 0.6(0.43,0.84) | 0.003 | 0.63(0.45,0.89) | 0.01 | 0.63(0.43,0.91) | 0.01 | 0.65(0.44,0.94) | 0.02 |
| Q3 | 92 / 122 | 0.45(0.32,0.64) | <0.001 | 0.48(0.33,0.69) | <0.001 | 0.55(0.37,0.81) | 0.003 | 0.54(0.36,0.81) | 0.003 |
| Q4 | 56 / 123 | 0.27(0.18,0.40) | <0.001 | 0.28(0.19,0.41) | <0.001 | 0.28(0.17,0.44) | <0.001 | 0.25(0.15,0.40) | <0.001 |
| P for trend |  |  | <0.001 |  | <0.001 |  | <0.001 |  | <0.001 |
| Per SD increment |  | 0.61(0.53,0.70) | <0.001 | 0.62(0.53,0.72) | <0.001 | 0.64(0.54,0.77) | <0.001 | 0.62(0.52,0.74) | <0.001 |
| **Betaine** |  |  |  |  |  |  |  |  |  |
| Q1 | 121 / 123 | 1(reference) |  | 1(reference) |  | 1(reference) |  | 1(reference) |  |
| Q2 | 157 / 124 | 1.29(0.91,1.82) | 0.15 | 1.22(0.86,1.73) | 0.27 | 1.19(0.83,1.70) | 0.35 | 1.38(0.94,2.03) | 0.10 |
| Q3 | 108 / 121 | 0.91(0.63,1.30) | 0.60 | 0.89(0.62,1.29) | 0.55 | 0.86(0.59,1.25) | 0.43 | 1.12(0.74,1.68) | 0.60 |
| Q4 | 105 / 123 | 0.87(0.60,1.25) | 0.44 | 0.79(0.54,1.14) | 0.21 | 0.75(0.51,1.10) | 0.14 | 1.18(0.76,1.83) | 0.47 |
| P for trend |  |  | 0.17 |  | 0.08 |  | 0.05 |  | 0.71 |
| Per SD increment |  | 0.92(0.81,1.04) | 0.19 | 0.89(0.78,1.02) | 0.09 | 0.88(0.77,1.00) | 0.05 | 1(0.86,1.17) | 0.96 |

**Abbreviation:** Q, quartile; OR, odds ratio; CI, confidence interval; BMI, body mass index; GDM, gestational diabetes mellitus;

Multivariable logistic regression models were used to estimate ORs and 95% CIs for the association between quartiles of individual choline subtypes and betaine intake and the odds of preeclampsia. The lowest quartile (Q1) was used as the reference group. P for trend was derived by modeling the median value of each quartile as a continuous variable. Per standard deviation (SD) increment estimates represent the change in odds of preeclampsia per one SD increase in the intake values.

Model 1 was adjusted for maternal age (years), gestational age at survey (weeks), and pre-pregnancy BMI (kg/m²).

Model 2 was further adjusted for socioeconomic and lifestyle factors, including monthly household income, educational attainment, physical activity (MET-hours/day), employment status, smoking status, alcohol consumption, and sleep quality.

Model 3 was additionally adjusted for dietary and reproductive variables, including daily energy intake (kcal/day), season of dietary assessment, parity, GDM, menstrual regularity, family history of hypertension, and supplement use (folic acid and multivitamins).

Model 4 further included psychological distress, specifically anxiety and depression scores, to account for potential confounding by maternal mental health status.

Two-sided *P* values are presented without adjustment for multiple comparisons, with *P* values below 0.001 reported as <0.001.

Supplementary Table 9. Sensitivity analysis excluding participants diagnosed with gestational diabetes mellitus (N = 419 pairs)

|  | Cases/controls | Model 1 | | Model 2 | | Model 3 | |
| --- | --- | --- | --- | --- | --- | --- | --- |
|  |  | OR (95% CI) | *P* value | OR (95% CI) | *P* value | OR (95% CI) | *P* value |
| **Total choline intake** | |  |  |  |  |  |  |
| Q1 | 174 / 105 | 1(reference) |  | 1(reference) |  | 1(reference) |  |
| Q2 | 114 / 105 | 0.71(0.49,1.03) | 0.07 | 0.74(0.51,1.08) | 0.12 | 0.83(0.56,1.24) | 0.36 |
| Q3 | 70 / 104 | 0.43(0.29,0.64) | <0.001 | 0.45(0.30,0.67) | <0.001 | 0.54(0.34,0.84) | 0.01 |
| Q4 | 61 / 105 | 0.37(0.25,0.56) | <0.001 | 0.37(0.25,0.56) | <0.001 | 0.46(0.28,0.78) | 0.004 |
| P for trend |  |  | <0.001 |  | <0.001 |  | <0.001 |
| Per SD increment |  | 0.61(0.52,0.71) | <0.001 | 0.62(0.53,0.72) | <0.001 | 0.64(0.52,0.78) | <0.001 |
| **Free choline** |  |  |  |  |  |  |  |
| Q1 | 163 / 105 | 1(reference) |  | 1(reference) |  | 1(reference) |  |
| Q2 | 91 / 105 | 0.56(0.38,0.81) | 0.002 | 0.57(0.39,0.83) | 0.004 | 0.59(0.40,0.87) | 0.01 |
| Q3 | 105 / 104 | 0.65(0.45,0.94) | 0.02 | 0.67(0.46,0.97) | 0.03 | 0.69(0.47,1.00) | 0.05 |
| Q4 | 60 / 105 | 0.37(0.25,0.55) | <0.001 | 0.37(0.25,0.56) | <0.001 | 0.36(0.24,0.55) | <0.001 |
| P for trend |  |  | <0.001 |  | <0.001 |  | <0.001 |
| Per SD increment |  | 0.7(0.60,0.81) | <0.001 | 0.7(0.60,0.81) | <0.001 | 0.69(0.59,0.81) | <0.001 |
| **Phosphocholine** |  |  |  |  |  |  |  |
| Q1 | 174 / 105 | 1(reference) |  | 1(reference) |  | 1(reference) |  |
| Q2 | 93 / 106 | 0.55(0.38,0.80) | 0.002 | 0.58(0.40,0.85) | 0.01 | 0.62(0.41,0.93) | 0.02 |
| Q3 | 93 / 103 | 0.59(0.40,0.86) | 0.01 | 0.59(0.40,0.87) | 0.01 | 0.63(0.41,0.97) | 0.03 |
| Q4 | 59 / 105 | 0.35(0.24,0.53) | <0.001 | 0.37(0.24,0.56) | <0.001 | 0.44(0.26,0.75) | 0.003 |
| P for trend |  |  | <0.001 |  | <0.001 |  | 0.003 |
| Per SD increment |  | 0.67(0.57,0.78) | <0.001 | 0.67(0.57,0.79) | <0.001 | 0.71(0.57,0.89) | 0.003 |
| **Glycerophosphocholine** | |  |  |  |  |  |  |
| Q1 | 177 / 107 | 1(reference) |  | 1(reference) |  | 1(reference) |  |
| Q2 | 93 / 103 | 0.57(0.39,0.83) | 0.004 | 0.6(0.41,0.89) | 0.01 | 0.65(0.43,0.99) | 0.04 |
| Q3 | 90 / 104 | 0.54(0.37,0.78) | 0.001 | 0.56(0.38,0.83) | 0.004 | 0.62(0.40,0.96) | 0.03 |
| Q4 | 59 / 105 | 0.37(0.25,0.56) | <0.001 | 0.38(0.25,0.58) | <0.001 | 0.52(0.30,0.90) | 0.02 |
| P for trend |  |  | <0.001 |  | <0.001 |  | 0.01 |
| Per SD increment |  | 0.63(0.53,0.74) | <0.001 | 0.63(0.53,0.75) | <0.001 | 0.67(0.52,0.86) | 0.001 |
| **Phosphatidylcholine** | |  |  |  |  |  |  |
| Q1 | 187 / 105 | 1(reference) |  | 1(reference) |  | 1(reference) |  |
| Q2 | 81 / 105 | 0.46(0.32,0.68) | <0.001 | 0.49(0.33,0.72) | <0.001 | 0.55(0.36,0.82) | 0.004 |
| Q3 | 91 / 104 | 0.52(0.36,0.75) | <0.001 | 0.54(0.37,0.79) | 0.002 | 0.6(0.40,0.90) | 0.01 |
| Q4 | 60 / 105 | 0.34(0.23,0.51) | <0.001 | 0.34(0.23,0.52) | <0.001 | 0.43(0.27,0.69) | <0.001 |
| P for trend |  |  | <0.001 |  | <0.001 |  | <0.001 |
| Per SD increment |  | 0.66(0.57,0.76) | <0.001 | 0.66(0.57,0.77) | <0.001 | 0.71(0.60,0.85) | <0.001 |
| **Sphingomyelin** |  |  |  |  |  |  |  |
| Q1 | 188 / 105 | 1(reference) |  | 1(reference) |  | 1(reference) |  |
| Q2 | 98 / 105 | 0.56(0.39,0.81) | 0.002 | 0.59(0.41,0.87) | 0.01 | 0.61(0.41,0.91) | 0.01 |
| Q3 | 86 / 104 | 0.49(0.34,0.72) | <0.001 | 0.51(0.35,0.76) | <0.001 | 0.58(0.38,0.89) | 0.01 |
| Q4 | 47 / 105 | 0.27(0.17,0.41) | <0.001 | 0.27(0.17,0.42) | <0.001 | 0.27(0.16,0.45) | <0.001 |
| P for trend |  |  | <0.001 |  | <0.001 |  | <0.001 |
| Per SD increment |  | 0.59(0.50,0.69) | <0.001 | 0.59(0.51,0.70) | <0.001 | 0.62(0.51,0.74) | <0.001 |
| **Betaine** |  |  |  |  |  |  |  |
| Q1 | 97 / 105 | 1(reference) |  | 1(reference) |  | 1(reference) |  |
| Q2 | 143 / 105 | 1.43(0.98,2.10) | 0.07 | 1.4(0.95,2.07) | 0.09 | 1.58(1.05,2.38) | 0.03 |
| Q3 | 89 / 104 | 0.91(0.61,1.37) | 0.66 | 0.89(0.59,1.35) | 0.59 | 1.21(0.77,1.89) | 0.40 |
| Q4 | 90 / 105 | 0.87(0.58,1.30) | 0.50 | 0.82(0.54,1.25) | 0.36 | 1.31(0.81,2.11) | 0.27 |
| P for trend |  |  | 0.16 |  | 0.1 |  | 0.5 |
| Per SD increment |  | 0.88(0.76,1.01) | 0.07 | 0.87(0.75,1.00) | 0.05 | 1.02(0.86,1.20) | 0.85 |

**Abbreviation:** Q, quartile; OR, odds ratio; CI, confidence interval; BMI, body mass index; GDM, gestational diabetes mellitus;

Multivariable logistic regression models were used to estimate ORs and 95% CIs for the associations between quartiles of individual choline subtypes and betaine intake and the odds of preeclampsia. This sensitivity analysis excluded participants with diagnosed GDM to assess the robustness of the associations independent of gestational diabetes. The lowest quartile (Q1) was used as the reference group. P for trend was derived by modeling the median value of each quartile as a continuous variable. Per standard deviation (SD) increment estimates represent the change in odds of preeclampsia per one SD increase in the intake values.

Model 1 was adjusted for maternal age (years), gestational age at survey (weeks), and pre-pregnancy BMI (kg/m²).

Model 2 was further adjusted for socioeconomic and lifestyle factors, including monthly household income, educational attainment, physical activity (MET-hours/day), employment status, smoking status, alcohol consumption, and sleep quality.

Model 3 was additionally adjusted for dietary and reproductive variables, including daily energy intake (kcal/day), season of dietary assessment, parity, GDM, menstrual regularity, family history of hypertension, and supplement use (folic acid and multivitamins).

Two-sided *P* values are presented without adjustment for multiple comparisons, with *P* values below 0.001 reported as <0.001.

Supplementary Table 10. Sensitivity analysis of associations between choline intake and preeclampsia, irrespective of total energy intake

|  | Cases/controls | Model 3 | | Adjusted model | |
| --- | --- | --- | --- | --- | --- |
|  |  | OR (95% CI) | *P* value | OR (95% CI) | *P* value |
| **Total choline intake** | |  |  |  |  |
| Q1 | 206 / 123 | 1(reference) |  | 1(reference) |  |
| Q2 | 133 / 123 | 0.7(0.50,0.98) | 0.04 | 0.76(0.53,1.10) | 0.15 |
| Q3 | 82 / 122 | 0.43(0.30,0.62) | <0.001 | 0.51(0.34,0.75) | <0.001 |
| Q4 | 70 / 123 | 0.36(0.25,0.53) | <0.001 | 0.37(0.25,0.56) | <0.001 |
| P for trend |  |  | <0.001 |  | <0.001 |
| Per SD increment |  | 0.65(0.56,0.75) | <0.001 | 0.67(0.57,0.78) | <0.001 |
| **Free choline** |  |  |  |  |  |
| Q1 | 192 / 123 | 1(reference) |  | 1(reference) |  |
| Q2 | 110 / 123 | 0.57(0.41,0.81) | 0.001 | 0.61(0.42,0.88) | 0.01 |
| Q3 | 116 / 122 | 0.61(0.43,0.86) | 0.004 | 0.65(0.44,0.94) | 0.02 |
| Q4 | 73 / 123 | 0.38(0.26,0.55) | <0.001 | 0.41(0.27,0.61) | <0.001 |
| P for trend |  |  | <0.001 |  | <0.001 |
| Per SD increment |  | 0.71(0.62,0.81) | <0.001 | 0.74(0.64,0.86) | <0.001 |
| **Phosphocholine** |  |  |  |  |  |
| Q1 | 204 / 123 | 1(reference) |  | 1(reference) |  |
| Q2 | 115 / 123 | 0.59(0.42,0.83) | 0.003 | 0.67(0.46,0.97) | 0.03 |
| Q3 | 103 / 122 | 0.55(0.39,0.78) | <0.001 | 0.52(0.35,0.76) | <0.001 |
| Q4 | 69 / 123 | 0.36(0.25,0.52) | <0.001 | 0.38(0.25,0.57) | <0.001 |
| P for trend |  |  | <0.001 |  | <0.001 |
| Per SD increment |  | 0.69(0.60,0.80) | <0.001 | 0.7(0.60,0.82) | <0.001 |
| **Glycerophosphocholine** | |  |  |  |  |
| Q1 | 200 / 123 | 1(reference) |  | 1(reference) |  |
| Q2 | 122 / 123 | 0.64(0.46,0.90) | 0.01 | 0.67(0.47,0.97) | 0.03 |
| Q3 | 100 / 122 | 0.52(0.36,0.74) | <0.001 | 0.54(0.36,0.79) | 0.001 |
| Q4 | 69 / 123 | 0.38(0.26,0.56) | <0.001 | 0.41(0.28,0.62) | <0.001 |
| P for trend |  |  | <0.001 |  | <0.001 |
| Per SD increment |  | 0.66(0.57,0.77) | <0.001 | 0.69(0.58,0.81) | <0.001 |
| **Phosphatidylcholine** | |  |  |  |  |
| Q1 | 229 / 123 | 1(reference) |  | 1(reference) |  |
| Q2 | 90 / 123 | 0.42(0.30,0.60) | <0.001 | 0.48(0.33,0.70) | <0.001 |
| Q3 | 101 / 122 | 0.47(0.33,0.66) | <0.001 | 0.5(0.34,0.73) | <0.001 |
| Q4 | 71 / 123 | 0.33(0.23,0.48) | <0.001 | 0.34(0.23,0.51) | <0.001 |
| P for trend |  |  | <0.001 |  | <0.001 |
| Per SD increment |  | 0.69(0.59,0.79) | <0.001 | 0.71(0.61,0.82) | <0.001 |
| **Sphingomyelin** |  |  |  |  |  |
| Q1 | 220 / 123 | 1(reference) |  | 1(reference) |  |
| Q2 | 123 / 123 | 0.6(0.43,0.84) | 0.003 | 0.62(0.43,0.89) | 0.01 |
| Q3 | 92 / 122 | 0.45(0.32,0.64) | <0.001 | 0.52(0.36,0.77) | <0.001 |
| Q4 | 56 / 123 | 0.27(0.18,0.40) | <0.001 | 0.25(0.17,0.39) | <0.001 |
| P for trend |  |  | <0.001 |  | <0.001 |
| Per SD increment |  | 0.61(0.53,0.70) | <0.001 | 0.62(0.53,0.73) | <0.001 |
| **Betaine** |  |  |  |  |  |
| Q1 | 121 / 123 | 1(reference) |  | 1(reference) |  |
| Q2 | 157 / 124 | 1.29(0.91,1.82) | 0.15 | 1.3(0.89,1.90) | 0.18 |
| Q3 | 108 / 121 | 0.91(0.63,1.30) | 0.60 | 1(0.67,1.50) | 1.00 |
| Q4 | 105 / 123 | 0.87(0.60,1.25) | 0.44 | 0.9(0.60,1.36) | 0.62 |
| P for trend |  |  | 0.17 |  | 0.37 |
| Per SD increment |  | 0.92(0.81,1.04) | 0.19 | 0.91(0.79,1.05) | 0.20 |

**Abbreviation:** Q, quartile; OR, odds ratio; CI, confidence interval; BMI, body mass index; GDM, gestational diabetes mellitus;

Multivariable logistic regression models were used to estimate ORs and 95% CIs for the associations between quartiles of individual choline subtypes and betaine intake and the odds of preeclampsia. The lowest quartile (Q1) was used as the reference group. P for trend was derived by modeling the median value of each quartile as a continuous variable. Per standard deviation (SD) increment estimates represent the change in odds of preeclampsia per one SD increase in the intake values.

Model 3 was adjusted for maternal age (years), gestational age at survey (weeks), pre-pregnancy BMI (kg/m²), monthly household income, educational attainment, physical activity (MET-hours/day), employment status, smoking status, alcohol consumption, sleep quality, daily energy intake (kcal/day), season of dietary assessment, parity, GDM, menstrual regularity, family history of hypertension, and supplement use (folic acid and multivitamins).

Adjusted model included the same covariates as Model 3, except that total daily energy intake was not included in the adjustment to examine the robustness of associations independent of energy standardization.

Two-sided *P* values are presented without adjustment for multiple comparisons, with *P* values below 0.001 reported as <0.001.

Supplementary Table 11. Sensitivity analysis of associations between residual energy-adjusted choline intake and preeclampsia odds

|  | Cases/controls | Model 1 | | Model 2 | | Model 3 | |
| --- | --- | --- | --- | --- | --- | --- | --- |
|  |  | OR (95% CI) | *P* value | OR (95% CI) | *P* value | OR (95% CI) | *P* value |
| **Total choline intake** | |  |  |  |  |  |  |
| Q1 | 206 / 123 | 1(reference) |  | 1(reference) |  | 1(reference) |  |
| Q2 | 133 / 123 | 0.7(0.50,0.98) | 0.04 | 0.74(0.52,1.04) | 0.08 | 0.79(0.54,1.14) | 0.21 |
| Q3 | 82 / 122 | 0.43(0.30,0.62) | <0.001 | 0.46(0.32,0.67) | <0.001 | 0.54(0.36,0.82) | 0.004 |
| Q4 | 70 / 123 | 0.36(0.25,0.53) | <0.001 | 0.37(0.25,0.54) | <0.001 | 0.42(0.26,0.68) | <0.001 |
| P for trend |  |  | <0.001 |  | <0.001 |  | <0.001 |
| Per SD increment |  | 0.65(0.56,0.75) | <0.001 | 0.66(0.57,0.76) | <0.001 | 0.69(0.57,0.84) | <0.001 |
| **Free choline** |  |  |  |  |  |  |  |
| Q1 | 192 / 123 | 1(reference) |  | 1(reference) |  | 1(reference) |  |
| Q2 | 110 / 123 | 0.57(0.41,0.81) | 0.001 | 0.59(0.42,0.84) | 0.003 | 0.62(0.43,0.88) | 0.01 |
| Q3 | 116 / 122 | 0.61(0.43,0.86) | 0.004 | 0.63(0.45,0.89) | 0.01 | 0.65(0.46,0.93) | 0.02 |
| Q4 | 73 / 123 | 0.38(0.26,0.55) | <0.001 | 0.39(0.27,0.57) | <0.001 | 0.39(0.27,0.57) | <0.001 |
| P for trend |  |  | <0.001 |  | <0.001 |  | <0.001 |
| Per SD increment |  | 0.71(0.62,0.81) | <0.001 | 0.72(0.62,0.83) | <0.001 | 0.72(0.62,0.83) | <0.001 |
| **Phosphocholine** |  |  |  |  |  |  |  |
| Q1 | 204 / 123 | 1(reference) |  | 1(reference) |  | 1(reference) |  |
| Q2 | 115 / 123 | 0.59(0.42,0.83) | 0.003 | 0.64(0.45,0.91) | 0.01 | 0.69(0.48,1.01) | 0.06 |
| Q3 | 103 / 122 | 0.55(0.39,0.78) | <0.001 | 0.57(0.40,0.82) | 0.002 | 0.56(0.38,0.85) | 0.01 |
| Q4 | 69 / 123 | 0.36(0.25,0.52) | <0.001 | 0.39(0.26,0.57) | <0.001 | 0.45(0.27,0.73) | 0.001 |
| P for trend |  |  | <0.001 |  | <0.001 |  | <0.001 |
| Per SD increment |  | 0.69(0.60,0.80) | <0.001 | 0.71(0.61,0.83) | <0.001 | 0.75(0.61,0.92) | 0.01 |
| **Glycerophosphocholine** | |  |  |  |  |  |  |
| Q1 | 200 / 123 | 1(reference) |  | 1(reference) |  | 1(reference) |  |
| Q2 | 122 / 123 | 0.64(0.46,0.90) | 0.01 | 0.69(0.49,0.98) | 0.04 | 0.71(0.49,1.04) | 0.08 |
| Q3 | 100 / 122 | 0.52(0.36,0.74) | <0.001 | 0.55(0.38,0.79) | 0.001 | 0.59(0.39,0.89) | 0.01 |
| Q4 | 69 / 123 | 0.38(0.26,0.56) | <0.001 | 0.4(0.27,0.59) | <0.001 | 0.5(0.30,0.83) | 0.01 |
| P for trend |  |  | <0.001 |  | <0.001 |  | 0.004 |
| Per SD increment |  | 0.66(0.57,0.77) | <0.001 | 0.68(0.58,0.79) | <0.001 | 0.72(0.58,0.90) | 0.003 |
| **Phosphatidylcholine** | |  |  |  |  |  |  |
| Q1 | 229 / 123 | 1(reference) |  | 1(reference) |  | 1(reference) |  |
| Q2 | 90 / 123 | 0.42(0.30,0.60) | <0.001 | 0.45(0.31,0.64) | <0.001 | 0.5(0.34,0.73) | <0.001 |
| Q3 | 101 / 122 | 0.47(0.33,0.66) | <0.001 | 0.5(0.35,0.71) | <0.001 | 0.55(0.37,0.80) | 0.002 |
| Q4 | 71 / 123 | 0.33(0.23,0.48) | <0.001 | 0.33(0.23,0.49) | <0.001 | 0.4(0.26,0.61) | <0.001 |
| P for trend |  |  | <0.001 |  | <0.001 |  | <0.001 |
| Per SD increment |  | 0.69(0.59,0.79) | <0.001 | 0.7(0.60,0.80) | <0.001 | 0.76(0.64,0.89) | <0.001 |
| **Sphingomyelin** |  |  |  |  |  |  |  |
| Q1 | 220 / 123 | 1(reference) |  | 1(reference) |  | 1(reference) |  |
| Q2 | 123 / 123 | 0.6(0.43,0.84) | 0.003 | 0.63(0.45,0.89) | 0.01 | 0.63(0.43,0.91) | 0.01 |
| Q3 | 92 / 122 | 0.45(0.32,0.64) | <0.001 | 0.48(0.33,0.69) | <0.001 | 0.55(0.37,0.81) | 0.003 |
| Q4 | 56 / 123 | 0.27(0.18,0.40) | <0.001 | 0.28(0.19,0.41) | <0.001 | 0.28(0.17,0.44) | <0.001 |
| P for trend |  |  | <0.001 |  | <0.001 |  | <0.001 |
| Per SD increment |  | 0.61(0.53,0.70) | <0.001 | 0.62(0.53,0.72) | <0.001 | 0.64(0.54,0.77) | <0.001 |
| **Betaine** |  |  |  |  |  |  |  |
| Q1 | 121 / 123 | 1(reference) |  | 1(reference) |  | 1(reference) |  |
| Q2 | 157 / 124 | 1.22(0.86,1.73) | 0.27 | 1.19(0.83,1.70) | 0.35 | 1.38(0.94,2.03) | 0.10 |
| Q3 | 108 / 121 | 0.89(0.62,1.29) | 0.55 | 0.86(0.59,1.25) | 0.43 | 1.12(0.74,1.68) | 0.60 |
| Q4 | 105 / 123 | 0.79(0.54,1.14) | 0.21 | 0.75(0.51,1.10) | 0.14 | 1.18(0.76,1.83) | 0.47 |
| P for trend |  |  | 0.08 |  | 0.05 |  | 0.71 |
| Per SD increment |  | 0.89(0.78,1.02) | 0.09 | 0.88(0.77,1.00) | 0.05 | 1(0.86,1.17) | 0.96 |

Supplementary Table 11. Associations between residual energy-adjusted choline intake and preeclampsia odds (sensitivity analysis)

**Abbreviation:** Q, quartile; OR, odds ratio; CI, confidence interval; BMI, body mass index; GDM, gestational diabetes mellitus;

This sensitivity analysis evaluated the associations between residual energy-adjusted intake of choline and betaine and the odds of preeclampsia. Intake values were adjusted for total energy using the residual method prior to quartile classification. Multivariable logistic regression models were used to estimate ORs and 95% CIs for the associations between quartiles of individual choline subtypes and betaine intake and the odds of preeclampsia. This sensitivity analysis excluded participants with diagnosed GDM to assess the robustness of the associations independent of gestational diabetes. The lowest quartile (Q1) was used as the reference group. P for trend was derived by modeling the median value of each quartile as a continuous variable. Per standard deviation (SD) increment estimates represent the change in odds of preeclampsia per one SD increase in the intake values.

Model 1 was adjusted for maternal age (years), gestational age at survey (weeks), and pre-pregnancy BMI (kg/m²).

Model 2 was further adjusted for socioeconomic and lifestyle factors, including monthly household income, educational attainment, physical activity (MET-hours/day), employment status, smoking status, alcohol consumption, and sleep quality.

Model 3 was additionally adjusted for dietary and reproductive variables, including daily energy intake (kcal/day), season of dietary assessment, parity, GDM, menstrual regularity, family history of hypertension, and supplement use (folic acid and multivitamins).

Two-sided *P* values are presented without adjustment for multiple comparisons, with *P* values below 0.001 reported as <0.001.
